# Supplementary material for: Data-centric AI approach for automated wildflower monitoring
Source: PLoS One. 2024 Sep 9;19(9):e0302958. doi: 10.1371/journal.pone.0302958 (PMC11383241; doi:10.1371/journal.pone.0302958)
Supplement: S1 Appendix — (DOCX) [file pone.0302958.s001.docx]

S1 Annex

Characteristics and statistics of wildflowers in EWD

Table 3 summarizes the EWD dataset characteristics and statistcs.

- The first column holds the Latin as well as common species name.
- The second column shows the defined floral count units (FCUs), and if deviating from the formal inflorescence type, the latter is indicated between square backets.
- The average number of individual flowers per FCU as well as the average number of FCUs per flowering plant is provided in the third column. The voucher ID of the digitized herbarium sheet from which these numbers are obtained, is included. All used herbarium sheets are part of the Naturalis Biodiversity Center collection. For FCUs that are nested inflorescences the average number of flowers is detailed with a multiplication of its factors between parentheses. For instance: the FCU of *Achillea millefolium*, specified as ‘corymb of capitula’, contains on average 1800 flowers, namely 150 capitula x 12 flowers per capitulum. In case the number of flowers per FCU is presented as a range, the mean values from the herbarium sheets are given between square brackets.
- The numbers of images on which a species occurs and total number of annotations per species are given in column four.
- The last column indicates the landscape types in which the species is found. The following abbreviations are used: R (roadside), U (urban green), C (cropland), G (weed-rich grassland), and M (marshland).

*Table 3: List of annotated EWD species.*

| ***Latin species name* \|**  **Common name** | **FCU [inflorescence]** | **# flowers/FCU**  **# FCU/plant voucher ID** | **# images**  **# ann** | **Habitat** |
| --- | --- | --- | --- | --- |
| *Achillea millefolium* \|  Yarrow | Corymb of capitula | 1800 (150x12)  1 WAG.1370471 | 110  1002 | R, U, C, G, M |
| *Achillea ptarmica* \|  Sneezewort | Corymb of capitula | 2100 (35x60) 1 U.1105540 | 26  378 | R, U, G, M |
| *Aegopodium podagraria* \|  Ground Elder | Compound umbel | 150 ([10-20]x10)  3 U.1083098 | 19  594 | R, U, C |
| *Agrostemma githago* \|  Common Corn-cockle | Single flower  [Dichasium] | 1  2-6 [4] WAG.1220195 | 2  2 | R |
| *Ajuga reptans* \|  Bugle | Verticillaster | 6  < 10 WAG.1614207 | 18  988 | G, M |
| *Alliaria petiolata* \|  Garlic Mustard | Axillary raceme | < 20  1-3 [2] L.3232265 | 19  489 | R, U |
| *Anchusa officinalis* \|  Common Bugloss | One-sided cyme | 10  10 L.3353555 | 26  1239 | U |
| *Anemone nemorosa* \|  Wood Anemone | Solitary | 1  1 L.4261872 | 19  1002 | R, U, M |
| *Angelica sylvestris* \|  Wild Angelica | Compound umbel | 625 (25x25)  20-30 [25] WAG.1238595 | 9  52 | R, G, M |
| *Anthemis tinctoria* \|  Golden Marguerite | Capitulum  [Corymb of capitula] | 225  20 L.3414430 | 8  140 | R, U, C |
| *Anthriscus sylvestris* \|  Cow Parsley | Compound umbel | 150 (15x10)  5 L.3342195 | 21  1559 | R, U, C, G, M |
| *Anthyllis vulneraria* \|  Kidney Vetch | Paired racemes | 20 (2x10)  1 L.3964932 | 20  495 | R, U |
| *Arctium lappa* \|  Greater Burdock | Capitulum | 40  < 100 [50] L.3414078 | 6  28 | R |
| *Argentina anserina* \| Silverweed | Solitary | 1  3 L.3250088 | 14  48 | R |
| *Baldellia ranunculoides* \|  Lesser Water-plantain | Single flower  [Thyrse] | 1  < 6 [5] L.2106363 | 11  216 | M |
| *Barbarea vulgaris* \|  Bittercress | Raceme | 50  1 L.3226727 | 2  44 | R, G |
| *Bellis perennis* \|  Daisy | Capitulum | 100 1 U.1116591 | 22  1429 | R, U, M |
| *Berteroa incana* \|  Hoary Alyssum | Raceme | 50  1 L.3964021 | 37  3177 | R, U |
| *Bidens frondosa* \|  Devil's Beggarticks | Capitulum | 10-100 [55]  10 L.3419866 | 3  17 | M |
| *Brassica rapa* \|  Turnip Rape | Raceme | 30  1 L.4453326 | 5  267 | R, G |
| *Butomus umbellatus* \|  Flowering Rush | Single terminal flower surrounded by 3 cymes | 20-25  1 WAG.1496417 | 1  3 | M |
| *Buttercup* (aggregate)* | Single flower | -- | 190  4196 | R, U, G, M |
| 1. *Ranunculus acris* \|  Meadow Buttercup | Single flower  [Cyme] | 1  < 20 [10] L.2071608 |  |  |
| 1. *Ranunculus repens* \| Creeping Buttercup | Single flower  [Cyme] | 1  1-3 [2] L.3217245 |  |  |
| *Cakile maritima* \|  Sea Rocket | Raceme | < 20 [10]  < 50 [25] L.3964029 | 2  31 | R |
| *Caltha palustris* \|  Marsh-marigold | Single flower  [Cyme] | 1 1-7 [4] L.4155790 | 50  1205 | G, M |
| *Calystegia sepium* \|  Hedge Bindweed | Solitary | 1  1-3 L.3364104 | 39  166 | R, U, G, M |
| *Cardamine pratensis* \|  Cuckooflower | Raceme | 10-20  1 L.3234986 | 155  1999 | G, M |
| *Centaurea cyanus* \| Cornflower | Capitulum  [Panicle of capitula] | 25  10 L.4468308 | 39  558 | R, U, C |
| *Centaurea jacea* \|  Brown Knapweed | Capitulum  [Corymb of capitula] | 50-100  < 5 L.2071371 | 32  493 | R, U, G |
| *Centaurea scabiosa* \| Greater Knapweed | Capitulum  [Cyme of capitula] | < 50  3-5 L.3427409 | 2  2 | R |
| *Centaurium erythraea* \|  Common Centaury | Single flower  [Cyme] | 1  12 L.3356311 | 2  101 | R |
| *Cerastium arvense* \|  Field Mouse-ear | Single flower  [Dichasium] | 1  5-15 [8] L.4232752 | 7  1735 | R |
| *Chamerion angustifolium* \| Fireweed | Raceme | < 50 [25] 1 L.3332652 | 1  12 | R |
| *Chamomile* (aggregate)* | Capitulum | -- | 45  2202 | R, U, C, M |
| 1. *Matricaria chamomilla* \| Chamomile 2. *Matricaria maritima* \|  Sea Mayweed | Capitulum  [Panicle of capitula]  Capitulum [Panicle of capitula] | 265 20 U.1136665 425  6 U.1136522 |  |  |
| *Chelidonium majus* \| Greater Celandine | Umbel | 4-5  6-40 [25] L.3235518 | 8  78 | R, U |
| *Cichorium intybus* \| Common Chicory | Panicle of capitula | 1000 (50x20)  1 L.3433148 | 13  265 | R |
| *Cicuta virosa* \| Northern Water Hemlock | Compound umbel | 1000 (10x100)  < 10 [5] L.3343708 | 5  20 | M |
| *Cirsium arvense* \|  Creeping Thistle | Capitulum  [Panicle of capitula] | < 100  32-69 [50] L.3431500 | 31  776 | R, G, M |
| *Cirsium dissectum* \| Meadow Thistle | Capitulum | 20-160 [70] 1 L.4346678 | 17  267 | M |
| *Cirsium palustre* \|  Marsh Thistle | Corymb of capitula | 500 (10x50)  1 L.3436572 | 29  110 | R, G, M |
| *Cirsium vulgare* \|  Spear Thistle | Capitulum  [Corymb of capitula] | 140-200 [170]  1-4 L.3436461 | 12  58 | R, G |
| *Convolvulus arvensis* \|  Field Bindweed | Single flower  [Cyme] | 1  10 (2x5) L.3363691 | 13  313 | R, U, G |
| *Crepis biennis* \|  Rough Hawksbeard | Capitulum  [Raceme of capitula] | 65 < 14 L.3420130 | 1  1 | R |
| *Crepis capillaris* \|  Smooth Hawksbeard | Capitulum  [Corymb of capitula] | < 60  < 100 L.3420724 | 17  474 | R, U, G |
| *Crithmum maritimum* \|  Rock Samphire | Compound umbel | 400 (20x20)  < 50 [25] WAG.1570171 | 2  144 | R |
| *Dactylorhiza maculata* \|  Heath Spotted Orchid | Spike | 10-50 [35]  1 WAG.1133347 | 29  825 | U, M |
| *Dactylorhiza praetermissa* \|  Southern Marsh Orchid | Spike | 20-80 [50]  1 L. 4304090 | 70  499 | G, M |
| *Daucus carota* \|  Wild Carrot | Compound umbel | 2500 (50x50)  1-3 [2] L.3343034 | 71  1302 | R, U, G |
| *Dianthus carthusianorum* \|  Carthusian Pink | Single flower  [Dichasium] | 1  1-8 [4] WAG.1314062 | 18  739 | R, U |
| *Dianthus deltoides* \|  Maiden Pink | Single flower [Cyme] | 1  20 L.3203245 | 5  546 | R, U |
| *Diplotaxis tenuifolia* \|  Wild Rocket | Branched raceme | < 30 [15]  1 L.3241246 | 1  59 | R |
| *Dipsacus fullonum* \|  Wild Teasel | Capitulum | 850  1-40 [20] WAG.1262841 | 1  3 | R |
| *Echium vulgare* \|  Viper's Bugloss | Raceme of one-sided cymes | 250 (25x10)  1 L.3352574 | 34  429 | R, U |
| *Epilobium hirsutum* \|  Great Willowherb | Single flower  [Raceme] | 1  10-20 [15] L.3324225 | 11  355 | R, M |
| *Epipactis helleborine* \|  Broad-leaved Helleborine | Spike | < 100 [30]  1 L.3165381 | 2  4 | R |
| *Erigeron annuus* \|  Annual Fleabane | Capitulum  [Corymb of capitula] | 200  5-50 [25] U.1118170 | 4  314 | R |
| *Erodium cicutarium* \| Redstem Filaree | Umbel | 3-8 [5]  1 L.3987260 | 17  710 | R, U |
| *Eryngium campestre* \|  Field Eryngo | Umbel of capitula | 3125 (25x125) 1 L.3335529 | 9  34 | R |
| *Eryngium maritimum* \|  Sea Holly | Capitulum | 25-50  5-10 L.3335326 | 5  132 | R |
| *Eschscholzia californica* \| California Poppy | Solitary | 1  1-5 [3] WAG.1145943 | 4  25 | U |
| *Eupatorium cannabinum*\| Hemp-agrimony | Corymb of capitula | 1750 (10x35x5) 1 L.3434525 | 31  417 | R, G, M |
| *Ficaria verna* \|  Lesser Celandine | Solitary | 1  1 L.4251183 | 31  818 | R, U, G, M |
| *Filipendula ulmaria* \| Meadowsweet | Compound coyrmb | < 500 [250]  1 L.2074516 | 26  161 | R, U, C, G, M |
| *Galeopsis tetrahit* \|  Common Hemp-nettle | Verticillaster | 30 1-3 [2] L.3354079 | 1  11 | G |
| *Gentiana pneumonanthe* \| Marsh Gentian | Cyme | < 10 [5]  1 WAG.1806697 | 2  5 | M |
| *Geranium molle* \|  Dove's-foot Crane's Bill | Two-flowered cyme | 2  10 L.3306450 | 28  289 | R, U |
| *Geranium pratense* \|  Meadow Crane's-bill | Single flower  [Raceme] | 1  5 L.3307515 | 2  40 | R |
| *Geranium robertianum* \|  Herb-Robert | Cyme | 1-2  < 10 L.4329197 | 2  21 | R |
| *Glebionis segetum* \|  Corn Marigold | Capitulum  [Corymb of capitula] | 165 15 L.4259655 | 6  195 | C |
| *Glechoma hederacea* \| Ground-ivy | Verticillaster | 2-5  1-4 L.4225684 | 15  510 | R, U, C, G |
| *Heracleum sphondylium* \|  Hogweed | Compound umbel | 200 (10x20) < 10 L.2071606 | 24  96 | R, G |
| *Hieracium umbellatum* \|  Canadian Hawkweed | Capitulum  [Panicle of capitula] | 55  50 L.3441447 | 12  515 | R, U |
| *Hottonia palustris* \|  Water Violet | Spike | 15 (3x5)  1 L.3361395 | 4  169 | M |
| *Hyacinthoides non-scripta* \| Bluebell | Spike | 5-10 [7]  1 L.2072439 | 5  69 | R, U |
| *Hypericum elodes* \|  Marsh St John's-wort | Single flower  [Cyme] | 1  6 L.4455652 | 12  838 | M |
| *Hypericum perforatum* \|  Perforate St John's-wort | Thyrse | 25-100 [50]  1 L.3322685 | 31  154 | R, U |
| *Hypochaeris radicata* \|  Flatweed | Capitulum | 20-30 [25]  < 9 WAG.1345380 | 35  602 | R, U, G, M |
| *Iris pseudacorus* \|  Yellow Flag | Monochasial cyme | 1-3 [2]  1 L.3153108 | 42  137 | R, C, G, M |
| *Jacobaea aquatica* \|  Water Ragwort | Corymb of capitula | 3500 (35x100)  1 L.3964730 | 3  18 | G |
| *Jacobaea paludosa* \|  Fen Ragwort | Corymb of capitula | 1200 (10x120)  1 L.4259490 | 13  20 | G |
| *Jacobaea vulgaris* \|  Ragwort | Corymb of capitula | 11250 (150x75)  1 L.2071228 | 36  125 | R, U, G |
| *Jasione montana* \|  Sheep's-bit | Cyme | < 100  5-10 L.3411637 | 16  373 | R, U |
| *Lamium album* \|  White Nettle | Verticillaster | 30 (5x6)  1 L.2071812 | 14  493 | R, U, G |
| *Lamium galeobdolon* \|  Yellow Archangel | Verticillaster | 30  1 U.1414561 | 8  48 | U |
| *Lamium purpureum* \|  Red Dead-nettle | Verticillaster | > 4  1-3 L.3964789 | 13  516 | R, U, G |
| *Leontodon saxatilis* \|  Lesser Hawkbit | Capitulum | > 25  < 10 L.3445976 | 2  16 | R |
| *Leucanthemum vulgare* \|  Ox-eye Daisy | Capitulum | 125  3-5 WAG.1516505 | 48  1429 | R, U, G |
| *Linaria vulgaris* \|  Common Toadflax | Raceme | < 20 [15]  1 U.1733958 | 16  250 | R, U |
| *Lotus corniculatus* \|  Bird's-foot Trefoil | Umbel-like cyme | 8  1-3 L.3295583 | 86  2238 | R, U, G, M |
| *Lysimachia thyrsiflora* \|  Tufted Loosestrife | Axillary raceme | < 20  < 10 L.3971465 | 5  89 | M |
| *Lysimachia vulgaris* \|  Yellow Loosestrife | Raceme | < 150 [100]  1 WAG.1222374 | 74  424 | R, U, G, M |
| *Lythrum salicaria* \|  Purple Loosestrife | Raceme | < 100 [50]  1 L.3331174 | 45  244 | R, U, G, M |
| *Malva alcea* \|  Greater Musk-mallow | Raceme | < 20 [10]  1 U.1373715 | 5  53 | R, U, G |
| *Malva moschata* \|  Musk Mallow | Raceme | < 10 [5]  1 L.3326089 | 6  72 | R, U |
| *Malva sylvestris* \| Common Mallow | Raceme | < 10 [5]  1 WAG.1079043 | 6  25 | R, U |
| *Melampyrum pratense* \|  Common Cow-wheat | One-sided raceme | < 10  < 10 L.2074388 | 2  175 | R |
| *Mentha aquatica* \|  Water Mint | Verticillaster | < 300 (3x100)  2-4 [3] L.3377866 | 24  263 | G, M |
| *Mimulus guttatus* \|  Yellow Monkeyflower | Cyme | 15-30 [15]  1 WAG.1184085 | 5  54 | R, M |
| *Myosotis arvensis* \|  Field Forgot-me-not | One-sided cyme | 5-15 [10]  1 L.3374012 | 2  66 | U |
| *Myosotis scorpioides* \|  True Forgot-me-not | One-sided cyme | < 20 [10]  < 10 [5] L.3371959 | 46  709 | G, M |
| *Narthecium ossifragum* \| *100* Bog Asphodel | Spike | 10-20  1 L.4226749 | 4  49 | M |
| *Neottia ovata* \|  Common Twayblade | Spike | 20-80 [35]  1 L.4271319 | 1  1 | M |
| *Nuphar lutea* \|  Yellow Water-lily | Solitary | 1  1 L.2073297 | 2  4 | M |
| *Nymphaea alba* \| White Waterlily | Solitary | 1  1-3 L.2073209 | 3  11 | M |
| *Oenanthe aquatica* \| Fine-leaved Water-dropwort | Compound umbel | 130 ([5-8]x20)  15 L.3336416 | 9  139 | M |
| *Oenothera glazioviana* \|  Large-flowered Evening-primrose | Raceme | < 100 1 L.2072221 | 22  70 | R, U, C, M |
| *Origanum vulgare* \| Oregano | Corymb of verticillasters | 300 (5x60)  1 L.3374416 | 3  59 | R |
| *Ornithogalum umbellatum* \|  Garden Star-of-Bethlehem | Raceme | 5-10 [7]  1 L.3149866 | 1  6 | U |
| *Papaver rhoeas* \|  Common Poppy | Solitary | 1  1 L.4170584 | 33  1367 | R, U, C |
| *Pastinaca sativa* \|  Parsnip | Compound umbel | 50 (5x10)  1 L.2577708 | 9  102 | R, G |
| *Pedicularis palustris* \|  Marsh Lousewort | Raceme | < 30 [15]  1 L.3388271 | 38  922 | G, M |
| *Pedicularis sylvatica* \|  Common Lousewort | Raceme | 4-6 [5]  1 L.3391435 | 8  200 | M |
| *Pentaglottis sempervirens* \|  Green Alkanet | One-sided cyme | < 15 [7]  < 10 [5] L.2072354 | 4  343 | R |
| *Persicaria amphibia* \| Longroot Smartweed | Thyrse | < 50  1-3 [2] L.3169963 | 6  21 | M |
| *Persicaria bistorta* \|  Bistort | Spike | 150  1 L.3174339 | 15  396 | R, C |
| *Petasites hybridus* \|  Butterbur | Spike of capitula | 1800 (60x30)  1 L. 3461555 | 3  79 | R, U |
| *Peucedanum palustre* \|  Milk Parsley | Compound umbel | 450 (15x30)  5 L.3334842 | 23  100 | M |
| *Picris hieracioides* \| Hawkweed Oxtongue | Capitulum  [Corymb of capitula] | 20-40 [30] 3-5 [4] L.3460857 | 4  28 | R |
| *Plantago lanceolata* \| Ribwort Plantain | Spike | < 100  < 10 L.3402373 | 149  1100 | R, U, C, G, M |
| *Platanthera bifolia* \|  Lesser Butterfly Orchid | Spike | 12-45 [20]  1 L.2079882 | 5  11 | M |
| *Polygonum persicaria* \|  Lady's Thumb | Thyrse | < 100  1-3 [2] U.1530781 | 5  306 | R, U, G, M |
| *Potentilla erecta* \|  Tormentil | Single flower  [Corymb] | 1  25 L.3239737 | 28  277 | M |
| *Potentilla indica* \|  Mock Strawberry | Solitary | 1 1-3 [2] L.3239631 | 1  1 | U |
| *Potentilla palustris* \|  Purple Marshlocks | Cyme | < 10  1 L.1902136 | 1  2 | M |
| *Potentilla recta* \|  Sulphur Cinquefoil | Single flower  [Corymb] | 1  5 L.2071894 | 2  48 | R |
| *Potentilla reptans* \| Creeping Cinquefoil | Solitary | 1  3-5 [4] L.3256888 | 5  15 | R |
| *Primula elatior* \|  Oxlip | One-sided drooping umbel | 1-13 [7] 1 L.3349709 | 25  127 | U, M |
| *Prunella vulgaris* \|  Common Self-heal | Verticillaster | < 20  1-3 L.3374128 | 10  189 | R |
| *Pulicaria dysenterica* \| Common Fleabane | Corymb of capitula | 1750 ([15-20]x100) 1 L.3460738 | 5  38 | R |
| *Ranunculus aquatilis* \| Common Water-crowfoot | Single flower  [Cyme] | 1 3 L.4200468 | 8  1410 | R, M |
| *Ranunculus flammula* \|  Lesser Spearwort | Single flower  [Thyrse] | 1 1-3 [2] L.4237081 | 52  566 | R, G, M |
| *Raphanus raphanistrum* \|  Wild Radish | Raceme | < 10  < 5 L.2073174 | 2  17 | U, C |
| *Rhinanthus angustifolius* \|  Narrow-leaved Rattle | Raceme | 10-30 [20]  1 L.3391878 | 35  583 | R, U, C, G, M |
| *Salvia nemorosa* \|  Woodland Sage | Verticillaster | 60-90 [75]  5-10 L.3381064 | 3  83 | U |
| *Salvia pratensis* \|  Meadow Clary | Verticillaster | < 80 (20x4)  3-5 L.3898352 | 11  297 | R |
| *Securigera varia* \|  Crownvetch | Raceme | 10-20 [15] 10-20 L.3282632 | 2  187 | R |
| *Senecio inaequidens* \|  Narrow-leaved Ragwort | Capitulum  [Corymb of capitula] | 100 < 100 L.3463486 | 23  374 | R, U, G |
| *Silene dioica* \|  Red Campion | Single flower  [Dichasium] | 1  12 (3x4) L.3207939 | 34  557 | R, U, C, G |
| *Silene flos-cuculi* \|  Ragged-robin | Dichasium | 10 1-2 L.4259230 | 48  325 | R, G, M |
| *Silene latifolia* \|  White Campion | Single flower  [Dichasium] | 1 15 L.3201711 | 20  296 | R, U, G |
| *Silene vulgaris* \|  Bladder Campion | Single flower  [Dichasium] | 1  20 L.3221973 | 25  581 | R, U |
| *Sium latifolium* \|  Great Water-parsnip | Compound umbel | 500 (30x20)  1 L.3345490 | 1  7 | M |
| *Solidago gigantea* \|  Tall Goldenrod | Panicle of capitula | 2000 (100x20)  1 L.3466501 | 11  218 | R, G |
| *Stachys palustris* \|  Marsh Woundwort | Verticillaster | 6 < 10 [3] L.3390656 | 2  13 | G, M |
| *Stellaria palustris* \|  Marsh Stitchwort | Single flower  [Two-branched cyme] | 1  20 L.2074015 | 25  361 | U, G, M |
| *Symphytum grandiflorum* \|  Hidcote Pink | One-sided cyme | < 10  3-5 L.4259871 | 1  66 | R |
| *Symphytum officinale* \|  Comfrey | One-sided cyme | < 15  < 10 L.4239119 | 28  261 | R, C, G, M |
| *Tanacetum vulgare* \|  Tansy | Corymb of capitula | 2500 (50x50)  1 L.3455627 | 37  586 | R, U, G |
| *Taraxacum officinale* \|  Dandelion | Capitulum | 200  1-3 L.3470645 | 33  352 | R, U, G |
| *Thalictrum flavum* \| Common Meadow-rue | Compound corymb | < 500 [250]  1 L.3237256 | 17  155 | G |
| *Thymus pulegioides* \|  Broad-leaved Thyme | Verticillaster | 10  < 10 WAG.1702398 | 1  180 | R |
| *Trifolium pratense* \|  Red Clover | Raceme | < 50  1-3 L.2071588 | 129  1309 | R, U, C, G, M |
| *Trifolium repens* \|  White Clover | Raceme | 30-50 [40]  1-3 [2] L.3310664 | 74  1765 | R, U, G, M |
| *Tussilago farfara* \|  Coltsfoot | Capitulum | 300  3-7 WAG.1426924 | 1  60 | U |
| *Valeriana dioica* \| Marsh Valerian | Compound panicle | 30-50 [40]  1 L. 3397586 | 5  322 | G, M |
| *Valeriana officinalis* \|  Valerian | Dichasial cyme | 200  1 L.3402961 | 22  230 | R, G, M |
| *Veronica chamaedrys* \|  Germander Speedwell | Raceme | 10  4 L.3393222 | 4  295 | R |
| *Veronica longifolia* \|  Garden Speedwell | Raceme | < 100 1-3 [2] U.1724234 | 9  124 | G |
| *Vicia cracca* \|  Tufted Vetch | Raceme | < 20  1-3 L.3289917 | 36  655 | R, U, C, G, M |
| *Vicia sativa* \|  Common Vetch | Raceme | < 20  1 L.3293548 | 33  181 | R, C, G, M |
| *Yellow Composite** | Capitulum | -- | 13  46 | R, M |
