# Supplementary material for: Data-centric AI approach for automated wildflower monitoring
Source: PLoS One. 2024 Sep 9;19(9):e0302958. doi: 10.1371/journal.pone.0302958 (PMC11383241; doi:10.1371/journal.pone.0302958)
Supplement: S1 File — (HTML) [file pone.0302958.s002.html]

 
 
  
 

 flowerpower  


 


 

     

 

 
     
     
     
      
 

 
 
 
 
  
  
   Flower Power 

 
 
 
 
 
 
 
 
  
  
 This notebook is the starting point for wildflower species detection and monitoring in large top view photos of aproximately 1m^2 patches of land using the Eindhoven Wildflower Dataset (EWD) collected and annotated by Gerard Schouten, DOI:  10.34894/U4VQJ6  containing images of 6720 x 4480 pixels. This notebook addresses several challenges while using these large images as train-validate-test data for transfer learning a F-RCNN object detection model. 
 Bas Michielsen MSc 

 
 
 
  
 
 
 
 
 In [ ]: 
 
      
     import   contextlib  ,   datetime  ,   glob  ,   io  ,   json  ,   math  ,   pandas  ,   os  ,   PIL  ,   random  ,   re  ,   seaborn  ,   shutil  ,   statistics  ,   torch 
 import   matplotlib.pyplot   as   plt 
 import   torchvision.models.detection   as   models 
 import   torchvision.transforms   as   transforms 
 from   torchvision.datasets   import   VisionDataset 
 from   torchvision.transforms.functional   import   to_tensor 
 import   torchvision.ops.boxes   as   bops 
 from   pvoc   import   load_pvoc_annotations 
 from   cocojamboo   import   coco_evaluator_class_metrics 

 print  (  "pandas version:"  ,   pandas  .  __version__  ) 
 print  (  "seaborn version:"  ,   seaborn  .  __version__  ) 
 print  (  "torch version:"  ,   torch  .  __version__  ) 
 print  (  "Is CUDA available?"  ,   torch  .  cuda  .  is_available  ()) 
  

      
 
 
 

 
 
 


 
 
    
      


 
 pandas version: 1.5.0
seaborn version: 0.12.2
torch version: 1.12.1+cu116
Is CUDA available? True
 
 
 

 

 

 
 
 
 
 
  
  
 🏗️ Set up ¶   Torchvision offers object detection reference scipts that can be used to get started quickly. This notebook uses those scripts and downloads them if they are not present yet. Also, a working folder named  output  is created, and a plotting function is defined. 

 
 
 
  
 
 
 
 
 In [ ]: 
 
      
     random_state   =   42 
 data_path   =   "data" 
 output_path   =   "output" 


 for   f   in   [  "coco_eval.py"  ,   "coco_utils.py"  ,   "engine.py"  ,   "transforms.py"  ,   "utils.py"  ]: 
     if   not   os  .  path  .  exists  (  f  ):   wget  .  download  (  "https://raw.githubusercontent.com/pytorch/vision/main/references/detection/"   +   f  ,   f  ) 
 from   engine   import   train_one_epoch  ,   evaluate 

 if   not   os  .  path  .  exists  (  output_path  ): 
     os  .  mkdir  (  output_path  ) 
 output_path   =   os  .  path  .  join  (  output_path  ,   datetime  .  datetime  .  now  ()  .  strftime  (  "%Y%m  %d  %H%M%S"  )) 
 if   not   os  .  path  .  exists  (  output_path  ): 
     os  .  mkdir  (  output_path  ) 

 def   barplot  (  df  ,   y  ,   x  ,   title  ,   palette  =  seaborn  .  color_palette  (  "deep"  ),   orient  =  "h"  ): 
     plt  .  figure  (  figsize  =  (  len  (  plot_data  )  /  7  ,  len  (  plot_data  )  /  6  )) 
     ax   =   seaborn  .  barplot  (  data  =  plot_data  ,   y  =  y  ,   x  =  x  ,   orient  =  orient  ,   palette  =  palette  ,   dodge  =  False  ) 
     ax  .  set_title  (  label  =  title  ,   loc  =  "left"  ) 
     ax  .  set_xlim  (  0  ,   1   +   int  (  plot_data  [  x  ]  .  max  ())   *   1.2  ) 
     ax  .  set_xlabel  (  str  (  int  (  plot_data  [  x  ]  .  sum  ()))   +   " "   +   ax  .  get_xlabel  ()) 
     for   i   in   ax  .  containers  : 
         ax  .  bar_label  (  i  ,) 

 def   parse_pycoco_metrics  (  epoch  ,   pycoco_train_metrics  ,   pycoco_eval_metrics  ): 
     record   =   dict  () 
     record  [  "epoch"  ]   =   epoch 
     for   name  ,   value   in   re  .  findall  (  '(\w+):[^(]+\(([^)]+)\)'  ,   pycoco_train_metrics  ): 
         if   name   ==   "lr"  :   continue   # exclude learning rate. 
         record  [  name  ]   =   float  (  value  ) 
     for   line   in   pycoco_eval_metrics  .  split  (  "  \n  "  ): 
         r   =   re  .  search  (  '[^(]+\(([^)]+)\) @\[ IoU=([^|]+)\| area=([^|]+)\| maxDets=([^\]]+)] = (-?\d+.\d+)'  ,   line  ) 
         if   not   r  :   continue 
         m  ,   iou  ,   area  ,   maxDets  ,   value   =   r  .  group  (  1  )  .  strip  (),   r  .  group  (  2  )  .  strip  ()  .  replace  (  "0."  ,   "."  ),   r  .  group  (  3  )  .  strip  (),   r  .  group  (  4  )  .  strip  (),   r  .  group  (  5  ) 
         metric   =   m 
         if   m   ==   "AP"  :   metric   =   "Average Precision" 
         if   m   ==   "AR"  :   metric   =   "Average Recall" 
         iou   =   " @"   +   iou   +   "IoU " 
         area   =   ""   if   area   ==   "all"   else   "("   +   area   +   ")" 
         maxDets   =   ""   if   maxDets   ==   "100"   else   "["   +   maxDets   +   "]"  
         name   =   metric   +   iou   +   area   +   maxDets 
         record  [  name  ]   =   float  (  value  ) 
     return   record 
  

      
 
 
 

 
 
 
 
 
  
  
 Load the dataset ¶   Look at a sample of the data, removing the PVOC features that are not going to be used. 

 
 
 
  
 
 
 
 
 In [ ]: 
 
      
     annotations   =   load_pvoc_annotations  (  data_path  ) 
 df   =   pandas  .  DataFrame  (  annotations  ) 
 df  .  drop  (  columns  =  [  "pose"  ,   "truncated"  ,   "difficult"  ,   "occluded"  ],   axis  =  1  ,   inplace  =  True  ) 
 df  .  sample  (  5  ) 
  

      
 
 
 

 
 
 


 
 
    
     Out[ ]: 


 
 
 
 
   
     
        
       filename 
       width 
       height 
       name 
       xmin 
       ymin 
       xmax 
       ymax 
     
   
   
     
       23301 
       20220511194147-Roadside.jpg 
       6720 
       4480 
       Leucanthemum vulgare 
       4034 
       335 
       4349 
       579 
     
     
       811 
       20210605142532-Cropland.jpg 
       6720 
       4480 
       Anthriscus sylvestris 
       4605 
       2592 
       4919 
       2871 
     
     
       28473 
       20220616201043-Roadside.jpg 
       6720 
       4480 
       Jasione montana 
       5847 
       1293 
       6058 
       1496 
     
     
       7808 
       20210524150415-Grassland.jpg 
       6720 
       4480 
       Buttercup * (aggregate) 
       623 
       697 
       746 
       800 
     
     
       57237 
       20210528061604-Marsh.jpg 
       6720 
       4480 
       Pedicularis palustris 
       3250 
       2124 
       3512 
       2468 
     
   
 
 
 

 

 

 

 
 
 
 
 
  
  
 ⚛️ Select a pretrained model ¶   Here the F-RCCN model that was pretrained with the  resnet50 feature pyramid network v2  backbone is selected as the base model. The ideal image size ( target_size ) can be specified for testing purposes, or is inferred from the model's transform  maximum  input size. The reason for selecting the maximum rather than the mean of the maximum and the minimum is that the minor quality loss as a result of downscaling input images is considered less than the quality loss as a result of cutting through bounding boxes when making tiles in the preprocessing step. So making the least number of tiles is preferred. Given the input image size of 6720 x 4480, using the maximum, and therefore accepting a small loss in resolution results in 15 tiles per input images, whereas using the mean of minimum and maximum (~1024) results in 24 tiles, greatly increasing the number of potentially sliced bounding boxes. 
 
  target_size  Ideal size for the input images, if 0 then it will be inferred from the selected model. 
 

 
 
 
  
 
 
 
 
 In [ ]: 
 
      
     target_size   =   0 


 model   =   models  .  fasterrcnn_resnet50_fpn_v2  (  weights  =  "DEFAULT"  ) 
 target_size   =   model  .  transform  .  max_size   if   target_size   ==   0   else   target_size 
 print  (  "The target_size for input images is"  ,   target_size  ) 
  

      
 
 
 

 
 
 


 
 
    
      


 
 The target_size for input images is 1333
 
 
 

 

 

 
 
 
 
 
  
  
 🎴 Preprocessing input images ¶   In case the input images are significantly larger than the input size for the model to train, and the bounding boxes for the objects are relatively small, the problem could arise that when the large input image is automatically resized, the objects inside the bounding boxes degrade greatly in resolution. Therefore, it is better to have input images that are closer to the correct input size for the model (target_size), and having bounding boxes that are relatively large in respect to the input images. A helper function is used to slice the input images into slices that are close to the target size. 
 
  wubble_margin  The number of pixels as a fraction of the slice width/height that a slicing line may move to become less destructive.  Coined by Gerard Schouten after been explained about the behaviour of how the line moves to the least destructive position "Oh, so it wubbles?", as in 'clumsily move from one side to the other' . 
  input_path  The path that the preprocessed images should be saved to. 
  data_file  The file containing all annotations after preprocessing. 
  force_preprocessing  If the file at  data_file  already exists, preprocessing will be skipped. Set this to True, to preprocess regardless. 
 

 
 
 
  
 
 
 
 
 In [ ]: 
 
      
     wubble_margin   =   .2 
 input_path   =   "input" 
 data_file   =   "data.csv" 
 force_preprocessing   =   False 

 def   partition  (  annotations  :   list  ,   size  :   int  ,   margin  :   float  ,   input_path  :   str  ,   output_path  :   str  ): 
     result   =   [] 
     line_margin   =   int  (  size   *   margin  ) 
     filenames   =   [] 
     for   obj   in   annotations  : 
         filename   =   obj  [  "filename"  ] 
         if   not   filename   in   filenames  : 
             filenames  .  append  (  filename  ) 
     for   filename   in   filenames  : 
         objects   =   [  x   for   x   in   annotations   if   x  [  "filename"  ]   ==   filename  ] 
         width  ,   height   =   objects  [  0  ][  "width"  ],   objects  [  0  ][  "height"  ] 
         image   =   PIL  .  Image  .  open  (  os  .  path  .  join  (  input_path  ,   filename  )) 
         filename  ,   fileext   =   os  .  path  .  splitext  (  filename  ) 
         vertical_num   =   int  (  width   /   size  ) 
         vertical_size   =   int  (  width   /   vertical_num  ) 
         num_vertical_lines   =   vertical_num   -  1 
         vertical_lines   =   [] 
         for   i   in   range  (  1  ,   num_vertical_lines   +  1  ): 
             line   =   i   *   vertical_size 
             line   =   _get_slice_line  (  line  ,   line_margin  ,   objects  ,   "xmin"  ,   "xmax"  ) 
             vertical_lines  .  append  (  line  ) 
         vertical_lines  .  append  (  width  ) 
         horizontal_num   =   int  (  height   /   size  ) 
         horizontal_size   =   int  (  height   /   horizontal_num  ) 
         num_horizontal_lines   =   horizontal_num   -  1 
         horizontal_lines   =   [] 
         for   i   in   range  (  1  ,   num_horizontal_lines   +  1  ): 
             line   =   i   *   horizontal_size 
             line   =   _get_slice_line  (  line  ,   line_margin  ,   objects  ,   "ymin"  ,   "ymax"  ) 
             horizontal_lines  .  append  (  line  ) 
         horizontal_lines  .  append  (  height  )   
         prev_v   =   0 
         prev_h   =   0 
         row   =   0 
         for   h   in   horizontal_lines  : 
             number   =   0 
             row   =   row   +   1 
             for   v   in   vertical_lines  : 
                 number   =   number   +  1 
                 new_filename   =   filename   +   "_"   +   _get_row_letter  (  row  )   +   str  (  number  )   +   fileext 
                 for   obj   in   [  x   for   x   in   objects   if   (  x  [  "xmin"  ]   <   v   and   x  [  "xmin"  ]   >   prev_v   and   x  [  "ymin"  ]   <   h   and   x  [  "ymin"  ]   >   prev_h  )   or   (  x  [  "xmax"  ]   <   v   and   x  [  "xmax"  ]   >   prev_v   and   x  [  "ymax"  ]   <   h   and   x  [  "ymax"  ]   >   prev_h  )]: 
                     if   obj  [  "xmin"  ]   <   prev_v   and   obj  [  "xmax"  ]   >   prev_v  : 
                         if   obj  [  "xmax"  ]   -   prev_v   >   int  ((  obj  [  "xmax"  ]   -   obj  [  "xmin"  ])  /  2  ): 
                             obj  [  "xmin"  ]   =   prev_v 
                         else  : 
                             continue 
                     if   obj  [  "xmin"  ]   <   v   and   obj  [  "xmax"  ]   >   v  : 
                         if   v   -   obj  [  "xmin"  ]   >   int  ((  obj  [  "xmax"  ]   -   obj  [  "xmin"  ])  /  2  ): 
                             obj  [  "xmax"  ]   =   v 
                         else  : 
                             continue 
                     if   obj  [  "ymin"  ]   <   prev_h   and   obj  [  "ymax"  ]   >   prev_h  : 
                         if   obj  [  "ymax"  ]   -   prev_h   >   int  ((  obj  [  "ymax"  ]   -   obj  [  "ymin"  ])  /  2  ): 
                             obj  [  "ymin"  ]   =   prev_h 
                         else  : 
                             continue 
                     if   obj  [  "ymin"  ]   <   h   and   obj  [  "ymax"  ]   >   h  : 
                         if   h   -   obj  [  "ymin"  ]   >   int  ((  obj  [  "ymax"  ]   -   obj  [  "ymin"  ])  /  2  ): 
                             obj  [  "ymax"  ]   =   h 
                         else  : 
                             continue 
                     obj  [  "filename"  ]   =   new_filename 
                     obj  [  "width"  ]   =   v   -   prev_v 
                     obj  [  "height"  ]   =   h   -   prev_h 
                     obj_w   =   obj  [  "xmax"  ]   -   obj  [  "xmin"  ] 
                     obj_h   =   obj  [  "ymax"  ]   -   obj  [  "ymin"  ] 
                     obj  [  "xmin"  ]   =   obj  [  "xmin"  ]   -   prev_v 
                     obj  [  "ymin"  ]   =   obj  [  "ymin"  ]   -   prev_h 
                     obj  [  "xmax"  ]   =   obj  [  "xmin"  ]   +   obj_w 
                     obj  [  "ymax"  ]   =   obj  [  "ymin"  ]   +   obj_h 
                     result  .  append  (  obj  ) 
                 tile   =   image  .  crop  ((  prev_v  ,   prev_h  ,   v  ,   h  )) 
                 tile  .  save  (  os  .  path  .  join  (  output_path  ,   new_filename  )) 
                 prev_v   =   v 
             prev_h   =   h 
             prev_v   =   0 
     return   result 

 def   _get_slice_line  (  mid  ,   margin  ,   objects  ,   min_index  ,   max_index  ): 
     if   margin   ==   0  : 
         return   mid 
     results   =   dict  () 
     for   d   in   range  (  margin  ): 
         for   delta   in   {  d  ,   d  *-  1  }: 
             line   =   mid   +   delta 
             count   =   sum  ([  1   for   obj   in   objects   if   (  obj  [  min_index  ]   <   line   and   obj  [  max_index  ]   >=   line  )]) 
             if   count   ==   0  :   # Return if a 'perfect' line is found. 
                 return   line 
             results  [  line  ]   =   count 
     return   min  (  zip  (  results  .  values  (),   results  .  keys  ()))[  1  ]   # Return the line with the least sliced objects. 

 def   _get_row_letter  (  row  ):  
     return   str  (  chr  (  ord  (  '`'  )  +  row  )) 

 if   force_preprocessing   or   not   os  .  path  .  exists  (  input_path  )   or   not   os  .  path  .  exists  (  os  .  path  .  join  (  input_path  ,   data_file  )): 
     annotations   =   load_pvoc_annotations  (  data_path  )  
     if   os  .  path  .  exists  (  input_path  ): 
         shutil  .  rmtree  (  input_path  ) 
     os  .  mkdir  (  input_path  ) 
     data   =   partition  (  annotations  ,   target_size  ,   wubble_margin  ,   data_path  ,   input_path  ) 
     pandas  .  DataFrame  (  data  )  .  to_csv  (  os  .  path  .  join  (  input_path  ,   "data.csv"  ),   index  =  False  ) 
 df   =   pandas  .  read_csv  (  os  .  path  .  join  (  input_path  ,   data_file  )) 
  

      
 
 
 

 
 
 
 
 
  
  
 🎆 Demonstrating a preprocessing result ¶   The following code demonstrates the result of the preprocessing step, given the filename of an original image. If no filename is given, it takes a random original image. This cell can be re-run to get a another random image. 
 
  demo_image  The filename of the original image for which the preprocessing result should be demonstrated. Can be left empty to take a random original image. 
 

 
 
 
  
 
 
 
 
 In [ ]: 
 
      
     demo_image   =   "" 


 if   demo_image   ==   ""  : 
     filenames   =   list  (  set  (  x  [  "filename"  ]   for   x   in   df  .  to_dict  (  "records"  ))) 
     demo_image   =   random  .  sample  (  filenames  ,   k  =  1  )[  0  ] 
     _  ,   ext   =   os  .  path  .  splitext  (  demo_image  ) 
     demo_image   =   os  .  path  .  join  (  data_path  ,   demo_image  [:  demo_image  .  rfind  (  "_"  )]  +  ext  ) 

 def   collect  (  path  :   str  ,   image_path  :   str  ): 
     columns   =   0 
     image_name  ,   ext   =   os  .  path  .  splitext  (  os  .  path  .  basename  (  image_path  )) 
     files   =   glob  .  glob  (  os  .  path  .  join  (  path  ,   image_name   +   "*"  )) 
     for   f   in   files  : 
         if   f  [  f  .  rfind  (  "_"  )  +  1  :][  0  ]   ==   "a"  :   columns   +=   1 
         else  :   continue 
     rows   =   int  (  len  (  files  )   /   columns  ) 
     ratios_w  ,   ratios_h  ,   images   =   [],   [],   [] 
     for   row   in   range  (  rows  ): 
         row   +=   1 
         for   col   in   range  (  columns  ): 
             col   +=   1 
             img   =   PIL  .  Image  .  open  (  os  .  path  .  join  (  path  ,   image_name   +   "_"   +   _get_row_letter  (  row  )   +   str  (  col  )   +   ext  )) 
             if   len  (  ratios_w  )   <   columns  :   ratios_w  .  append  (  img  .  width  ) 
             if   col   ==   0   or   col   %   columns   ==   0  :   ratios_h  .  append  (  img  .  height  ) 
             images  .  append  (  img  ) 
     return   images  ,   rows  ,   columns  ,   ratios_w  ,   ratios_h 

 print  (  "Image:"  ,   demo_image  ) 
 images  ,   rows  ,   columns  ,   ratios_w  ,   ratios_h   =   collect  (  input_path  ,   demo_image  ) 
 fig  ,   subplots   =   plt  .  subplots  (  nrows  =  rows  ,   ncols  =  columns  ,   figsize  =  (  25  ,  15  ),   gridspec_kw  =  {  "width_ratios"  :  ratios_w  ,   "height_ratios"  :  ratios_h  }) 
 subplots   =   subplots  .  flatten  () 
 for   i   in   range  (  len  (  images  )): 
     subplots  [  i  ]  .  set_xticks  ([]) 
     subplots  [  i  ]  .  set_yticks  ([]) 
     subplots  [  i  ]  .  imshow  (  images  [  i  ]) 
  

      
 
 
 

 
 
 


 
 
    
      


 
 Image: data/20210403170602-Grassland.jpg
 
 
 
 
    
      


 
 
 

 

 

 

 
 
 
 
 
  
  
 🔭 Observations overview ¶   An overview of the number of observations per label is presented below. 

 
 
 
  
 
 
 
 
 In [ ]: 
 
      
     plot_data   =   df  .  groupby  (  by  =  [  "name"  ])[  "width"  ]  .  count  ()  .  rename  (  "observations"  )  .  reset_index  ()  .  sort_values  (  by  =  "observations"  ,   ascending  =  False  ) 
 barplot  (  plot_data  ,   "name"  ,   "observations"  ,   "Observations per label"  ) 
  

      
 
 
 

 
 
 


 
 
    
      


 
 
 

 

 

 

 
 
 
 
 
  
  
 🪓 Train-Validate-Test split ¶   Due to the fact that in object detection a single image could contain observations for multiple objects as well as for different objects it is not easy to make a balanced data set. If a certain image is selected for the reason that it has an observation for one specific object it may collaterally include more objects. By providing the sample size for the three different datasets (train, validate, test) it is possible to calculate all permutations of possible solutions that bring to the exact numbers and then select one of those permutations randomly. However, this approach of precalculating all permutations beforehand takes exponentially more time when the dataset gets larger. Therefore, the function below takes a trial-and-error approach by making many attempts and stopping early when a solutions is found, which only takes linearly more time when the dataset gets larger. If no solution is found with a certain set of attempts, it starts removing rarer objects from the dataset and try again, until a perfect solution is found. It is thus technically possible that an earlier perfect solution does exist but is not found, a label gets removed, and then the function returns the next best perfect solution. However the likelihood for this to happen for a specific label is inverse to the number of observations for that label relative to the required sample size ( train_size  +  validate_size  +  test_size ). The function starts by immediately removing labels having a number of observations less than 110% of the required sample size and increases this margin with steps of 10% for every n attempts at finding a perfect solution. The value for n can be increased in order to be more parsimonious about removing labels, at the cost of increasing calculation time. 
 
  train_size  The number of observations per label in the train dataset. 
  validate_size  The number of observations per label in the validate dataset. 
  test_size  The number of observations per label in the test dataset. 
  n_attempts  The number of attempts to make per increment in margin. 
 

 
 
 
  
 
 
 
 
 In [ ]: 
 
      
     train_size   =   250 
 validate_size   =   50 
 test_size   =   50 
 n_attempts   =   9 


 def   random_items_with_sum  (  items  ,   target_sum  ): 
     if   target_sum   <=   0  :   return   None 
     for   _   in   range  (  len  (  items  .  keys  ())  *  1000  ):   # If a combination is not found after this many attempts, we assume it does not exist. It is a magic number, but we need some sort of escape. 
         k   =   random  .  randint  (  0  ,   len  (  items  .  keys  ())) 
         combination   =   random  .  sample  (  list  (  items  .  keys  ()),   k  =  k  ) 
         if   sum  ([  items  [  k  ]   for   k   in   combination  ])   ==   target_sum  : 
             return   dict  (((  key  ,   items  [  key  ])   for   key   in   combination  )) 
     return   None 

 def   balanced_train_validate_test_split  (  data  ,   train_size  ,   validate_size  ,   test_size  ,   n_attempts  ,   random_state  =  None  ): 
     import   random  ,   copy 
     if   random_state   !=   None  :   random  .  seed  (  random_state  ) 
     names   =   list  (  set  ([  x  [  "name"  ]   for   x   in   data  ])) 
     train  ,   validate  ,   test  ,   removed   =   [],   [],   [],   [] 
     groups   =   df  .  groupby  (  by  =  [  "name"  ,   "filename"  ])[  "width"  ]  .  count  ()  .  to_dict  () 
     rows   =   dict  () 
     for   k  ,   v   in   groups  .  items  ():   # Make a pool of files per class name and the number of times the class appears in that file. 
         name   =   k  [  0  ] 
         if   name   in   rows  :   rows  [  name  ][  k  [  1  ]]   =   v 
         else  :   rows  [  name  ]   =   {  k  [  1  ]:  v  } 
     margin   =   0.0 
     found   =   False 
     while   not   found  : 
         margin   +=   .05 
         for   _   in   range  (  n_attempts  ): 
             records   =   copy  .  deepcopy  (  rows  ) 
             train  ,   validate  ,   test  ,   removed   =   [],   [],   [],   [] 
             labels   =   sorted  (  records  ,   key  =  lambda   k  :   len  (  records  [  k  ])) 
             for   label   in   labels  : 
                 items   =   records  [  label  ] 
                 if   sum  (  items  .  values  ())   <   (  train_size   +   validate_size   +   test_size  )   *   (  1.0   +   margin  ): 
                     for   lbl   in   labels  : 
                         records  [  lbl  ]   =   dict  (((  key  ,   records  [  lbl  ][  key  ])   for   key   in   records  [  lbl  ]   if   key   not   in   items  .  keys  ())) 
                     removed  .  append  (  label  ) 
             labels   =   [  x   for   x   in   labels   if   x   not   in   removed  ] 
             labels  .  reverse  () 
             for   label   in   labels  : 
                 items   =   records  [  label  ] 
                 test_items   =   None 
                 test_size_for_label   =   test_size   -   len  ([  x   for   x   in   test   if   x  [  "name"  ]   ==   label  ]) 
                 if   test_size_for_label   >   0  : 
                     test_items   =   random_items_with_sum  (  items  ,   test_size_for_label  ) 
                     if   test_items   ==   None  :   break 
                 validate_items   =   None 
                 validate_size_for_label   =   validate_size   -   len  ([  x   for   x   in   validate   if   x  [  "name"  ]   ==   label  ]) 
                 if   validate_size_for_label   >   0  : 
                     validate_items   =   random_items_with_sum  (  items  ,   validate_size_for_label  ) 
                     if   validate_items   ==   None  :   break         
                 train_items   =   None 
                 train_size_for_label   =   train_size   -   len  ([  x   for   x   in   train   if   x  [  "name"  ]   ==   label  ]) 
                 if   train_size_for_label   >   0  : 
                     train_items   =   random_items_with_sum  (  items  ,   train_size_for_label  ) 
                     if   train_items   ==   None  :   break 
                 for   lbl   in   labels  : 
                     records  [  lbl  ]   =   dict  (((  key  ,   records  [  lbl  ][  key  ])   for   key   in   records  [  lbl  ]   if   key   not   in   items  .  keys  ())) 
                 if   test_items   !=   None  : 
                     test   +=   [  x   for   x   in   data   if   x  [  "filename"  ]   in   test_items  ] 
                 if   validate_items   !=   None  : 
                     validate   +=   [  x   for   x   in   data   if   x  [  "filename"  ]   in   validate_items  ] 
                 if   train_items   !=   None  : 
                     train   +=   [  x   for   x   in   data   if   x  [  "filename"  ]   in   train_items  ] 
             selected   =   len  (  names  )   -   len  (  removed  ) 
             found   =   (  len  (  test  )   ==   selected   *   test_size  )   and   (  len  (  validate  )   ==   selected   *   validate_size  )   and   (  len  (  train  )   ==   selected   *   train_size  ) 
             if   found  :  
                 break 
     return   train  ,   validate  ,   test  ,   removed 

 data   =   df  .  to_dict  (  "records"  ) 
 train_data  ,   validate_data  ,   test_data  ,   removed   =   balanced_train_validate_test_split  (  data  ,   train_size  ,   validate_size  ,   test_size  ,   n_attempts  ,   random_state  =  random_state  ) 
 print  (  "The train set has"  ,   len  (  train_data  ),   "observations."  ) 
 pandas  .  DataFrame  (  train_data  )  .  to_csv  (  os  .  path  .  join  (  output_path  ,   "train.csv"  ),   index  =  False  ) 
 print  (  "The validate set has"  ,   len  (  validate_data  ),   "observations."  ) 
 pandas  .  DataFrame  (  validate_data  )  .  to_csv  (  os  .  path  .  join  (  output_path  ,   "validate.csv"  ),   index  =  False  ) 
 print  (  "The test set has"  ,   len  (  test_data  ),   "observations."  ) 
 pandas  .  DataFrame  (  test_data  )  .  to_csv  (  os  .  path  .  join  (  output_path  ,   "test.csv"  ),   index  =  False  ) 
 print  (  "The following labels were removed:  \n  "  ,   removed  ) 

 plot_data   =   pandas  .  DataFrame  (  train_data  )  .  groupby  (  by  =  [  "name"  ])[  "width"  ]  .  count  ()  .  rename  (  "observations"  )  .  reset_index  ()  .  sort_values  (  by  =  [  "observations"  ,   "name"  ]) 
 barplot  (  plot_data  ,   "name"  ,   "observations"  ,   "Train dataset"  ) 

 plot_data   =   pandas  .  DataFrame  (  validate_data  )  .  groupby  (  by  =  [  "name"  ])[  "width"  ]  .  count  ()  .  rename  (  "observations"  )  .  reset_index  ()  .  sort_values  (  by  =  [  "observations"  ,   "name"  ]) 
 barplot  (  plot_data  ,   "name"  ,   "observations"  ,   "Validate dataset"  ) 

 plot_data   =   pandas  .  DataFrame  (  test_data  )  .  groupby  (  by  =  [  "name"  ])[  "width"  ]  .  count  ()  .  rename  (  "observations"  )  .  reset_index  ()  .  sort_values  (  by  =  [  "observations"  ,   "name"  ]) 
 barplot  (  plot_data  ,   "name"  ,   "observations"  ,   "Test dataset"  ) 
  

      
 
 
 

 
 
 


 
 
    
      


 
 The train set has 12250 observations.
The validate set has 2450 observations.
The test set has 2450 observations.
The following labels were removed:
 ['Crepis biennis', 'Neottia ovata', 'Potentilla indica', 'Agrostemma githago', 'Centaurea scabiosa', 'Dipsacus fullonum', 'Potentilla palustris', 'Butomus umbellatus', 'Gentiana pneumonanthe', 'Epipactis helleborine', 'Nuphar lutea', 'Ornithogalum umbellatum', 'Galeopsis tetrahit', 'Sium latifolium', 'Bidens frondosa', 'Chamerion angustifolium', 'Raphanus raphanistrum', 'Cakile maritima', 'Nymphaea alba', 'Platanthera bifolia', 'Stachys palustris', 'Arctium lappa', 'Origanum vulgare', 'Barbarea vulgaris', 'Diplotaxis tenuifolia', 'Leontodon saxatilis', 'Malva sylvestris', 'Persicaria amphibia', 'Tussilago farfara', 'Centaurium erythraea', 'Geranium robertianum', 'Jacobaea aquatica', 'Potentilla reptans', 'Thymus pulegioides', 'Cicuta virosa', 'Symphytum grandiflorum', 'Malva alcea', 'Picris hieracioides', 'Potentilla recta', 'Myosotis arvensis', 'Jacobaea paludosa', 'Eryngium campestre', 'Geranium pratense', 'Hyacinthoides non-scripta', 'Eschscholzia californica', 'Salvia nemorosa', 'Erigeron annuus', 'Pulicaria dysenterica', 'Crithmum maritimum', 'Securigera varia', 'Yellow Composite * (aggregate)', 'Malva moschata', 'Melampyrum pratense', 'Mimulus guttatus', 'Narthecium ossifragum', 'Petasites hybridus', 'Angelica sylvestris', 'Lamium galeobdolon', 'Lysimachia thyrsiflora', 'Argentina anserina', 'Polygonum persicaria', 'Cirsium vulgare', 'Pastinaca sativa', 'Hottonia palustris', 'Prunella vulgaris', 'Valeriana dioica', 'Chelidonium majus', 'Eryngium maritimum', 'Glebionis segetum', 'Anthemis tinctoria', 'Pentaglottis sempervirens', 'Oenanthe aquatica', 'Pedicularis sylvatica', 'Brassica rapa', 'Veronica chamaedrys', 'Primula elatior', 'Oenothera glazioviana', 'Heracleum sphondylium', 'Veronica longifolia', 'Cichorium intybus', 'Linaria vulgaris', 'Peucedanum palustre', 'Cirsium palustre', 'Baldellia ranunculoides', 'Jacobaea vulgaris', 'Senecio inaequidens', 'Solidago gigantea', 'Epilobium hirsutum', 'Silene latifolia', 'Symphytum officinale', 'Salvia pratensis', 'Vicia sativa', 'Hypericum perforatum', 'Convolvulus arvensis', 'Filipendula ulmaria', 'Thalictrum flavum', 'Jasione montana', 'Iris pseudacorus', 'Calystegia sepium', 'Potentilla erecta', 'Valeriana officinalis', 'Geranium molle', 'Cirsium dissectum', 'Stellaria palustris', 'Lythrum salicaria', 'Taraxacam officinale', 'Mentha aquatica', 'Achillea ptarmica', 'Silene flos-cuculi', 'Echium vulgare', 'Lysimachia vulgaris']
 
 
 
 
    
      


 
 
 

 
 
    
      


 
 
 

 
 
    
      


 
 
 

 

 

 

 
 
 
 
 
  
  
 🌷 Species ¶   An overview of the species that the model will be trained on. 

 
 
 
  
 
 
 
 
 In [ ]: 
 
      
     species   =   [  name   for   name   in   list  (  set  (  x  [  "name"  ]   for   x   in   test_data  ))] 
 random  .  shuffle  (  species  ) 
 classes   =   [  "__background__"  ]   +   species 
 model  .  roi_heads  .  box_predictor   =   models  .  faster_rcnn  .  FastRCNNPredictor  (  model  .  roi_heads  .  box_predictor  .  cls_score  .  in_features  ,   len  (  classes  ))     
 print  (  "The species that the model will be trained on are:"  ) 
 df_species   =   pandas  .  DataFrame  (  train_data  ) 
 df_species  [  "size"  ]   =   df_species  .  apply  (  lambda   x  :   (  x  [  "xmax"  ]   -   x  [  "xmin"  ])   *   (  x  [  "ymax"  ]   -   x  [  "ymin"  ]),   axis  =  1  ) 
 df_species   =   df_species  .  sort_values  (  "size"  ,   ascending  =  False  )  .  drop_duplicates  ([  "name"  ])  .  sort_values  (  "name"  ) 
 plot_data   =   df_species  .  to_dict  (  "records"  ) 
 rows   =   math  .  ceil  (  len  (  plot_data  )  /  4  ) 
 cols   =   4 
 fig  ,   subplots   =   plt  .  subplots  (  nrows  =  rows  ,   ncols  =  cols  ,   figsize  =  (  len  (  plot_data  ),   len  (  plot_data  )  *  1.5  )) 
 subplots   =   subplots  .  flatten  () 
 for   i  ,   file   in   enumerate  (  plot_data  ): 
     img   =   PIL  .  Image  .  open  (  os  .  path  .  join  (  input_path  ,   file  [  "filename"  ])) 
     img   =   img  .  crop  ((  file  [  "xmin"  ],   file  [  "ymin"  ],   file  [  "xmax"  ],   file  [  "ymax"  ])) 
     subplots  [  i  ]  .  imshow  (  img  ) 
     subplots  [  i  ]  .  set  (  title  =  file  [  "name"  ]) 
 for   i   in   range  (  rows   *   cols  ): 
     subplots  [  i  ]  .  set_xticks  ([]) 
     subplots  [  i  ]  .  set_yticks  ([]) 
  

      
 
 
 

 
 
 


 
 
    
      


 
 The species that the model will be trained on are:
 
 
 
 
    
      


 
 
 

 

 

 

 
 
 
 
 
  
  
 🗃️ Compose the data sets ¶   In order for Torchvision to read the data, three different DataSets needs to be composed. One of them is for the training data, a second for the validation data, and the last for the test data. 

 
 
 
  
 
 
 
 
 In [ ]: 
 
      
     class   Dataset  (  VisionDataset  ): 
     def   __init__  (  self  ,   root  ,   data  ,   classes  ,   transforms  =  None  ,   transform  =  None  ,   target_transform  =  None  ): 
         super  ()  .  __init__  (  root  ,   transforms  ,   transform  ,   target_transform  ) 
         self  .  classes   =   classes 
         self  .  images   =   glob  .  glob  (  os  .  path  .  join  (  self  .  root  ,   "*.jpg"  )) 
         self  .  data   =   data 
         images   =   [] 
         for   obj   in   data  : 
             filename   =   obj  [  "filename"  ] 
             if   not   filename   in   images  : 
                 images  .  append  (  filename  ) 
         self  .  images   =   images 

     def   __getitem__  (  self  ,   i  ): 
         image_path   =   os  .  path  .  join  (  self  .  root  ,   self  .  images  [  i  ]) 
         image   =   PIL  .  Image  .  open  (  image_path  )  .  convert  (  "RGB"  ) 
         objects  ,   labels   =   [],   [] 
         records   =   [  x   for   x   in   self  .  data   if   x  [  "filename"  ]   ==   self  .  images  [  i  ]] 
         for   record   in   records  : 
             objects  .  append  ([  int  (  record  [  "xmin"  ]),   int  (  record  [  "ymin"  ]),   int  (  record  [  "xmax"  ]),   int  (  record  [  "ymax"  ])]) 
             labels  .  append  (  self  .  classes  .  index  (  record  [  "name"  ])) 
         boxes   =   torch  .  as_tensor  (  objects  ,   dtype  =  torch  .  float32  ) 
         target   =   {} 
         target  [  "boxes"  ]   =   boxes 
         target  [  "labels"  ]   =   torch  .  as_tensor  (  labels  ) 
         target  [  "image_id"  ]   =   torch  .  tensor  ([  i  ]) 
         target  [  "area"  ]   =   (  boxes  [:,   3  ]   -   boxes  [:,   1  ])   *   (  boxes  [:,   2  ]   -   boxes  [:,  0  ])   if   len  (  boxes  )   >   0   else   torch  .  zeros  (  1  ) 
         target  [  "iscrowd"  ]   =   torch  .  zeros  ((  len  (  boxes  ),),   dtype  =  torch  .  int64  ) 
         if   self  .  transforms   is   not   None  : 
             image   =   self  .  transforms  (  image  ) 
         tensor   =   to_tensor  (  image  ) 
         return   tensor  ,   target 
    
     def   __len__  (  self  ): 
         return   len  (  self  .  images  ) 

 train_transforms   =   transforms  .  Compose  ([ 
         transforms  .  RandomAdjustSharpness  (  sharpness_factor  =  4  ), 
         transforms  .  RandomAutocontrast  () 
 ]) 

 train_dataset   =   Dataset  (  input_path  ,   train_data  ,   classes  ,   transforms  =  train_transforms  ) 
 print  (  "The train dataset contains"  ,   len  (  train_dataset  ),   "images."  ) 
 validate_dataset   =   Dataset  (  input_path  ,   validate_data  ,   classes  ) 
 print  (  "The validate dataset contains"  ,   len  (  validate_dataset  ),   "images."  ) 
 test_dataset   =   Dataset  (  input_path  ,   test_data  ,   classes  ) 
 print  (  "The test dataset contains"  ,   len  (  test_dataset  ),   "images."  ) 
  

      
 
 
 

 
 
 


 
 
    
      


 
 The train dataset contains 3012 images.
The validate dataset contains 642 images.
The test dataset contains 630 images.
 
 
 

 

 

 
 
 
 
 
  
  
 ♻️ Prepare data loaders and a maximum number of epochs ¶   A data loader arranges for the data in the dataset to be loaded into the memory of the system in batches. It is possible to increase the batch size if enough free memory is available. 
 
  batch_size  The number of images per step to load into memory. 
 

 
 
 
  
 
 
 
 
 In [ ]: 
 
      
     batch_size   =   6 


 def   collate  (  batch  ): 
     return   tuple  (  zip  (  *  batch  )) 
 train_loader   =   torch  .  utils  .  data  .  DataLoader  (  train_dataset  ,   batch_size  =  batch_size  ,   shuffle  =  True  ,   num_workers  =  0  ,   collate_fn  =  collate  ) 
 validate_loader   =   torch  .  utils  .  data  .  DataLoader  (  validate_dataset  ,   batch_size  =  1  ,   shuffle  =  False  ,   num_workers  =  0  ,   collate_fn  =  collate  ) 
 test_loader   =   torch  .  utils  .  data  .  DataLoader  (  test_dataset  ,   batch_size  =  1  ,   shuffle  =  False  ,   num_workers  =  0  ,   collate_fn  =  collate  ) 
  

      
 
 
 

 
 
 
 
 
  
  
 ▶️ Run training and gather validation metrics ¶   The object detection reference scripts from PyTorch vision will be used to run a train + validate loop. The functions  train_one_epoch  and  evaluate  are called repeatedly until the point of early stopping is reached, calculated by finding no more gain in validation performance. In this case an SGD optimizer with momentum is used, and the learning rate scheduler is instructed to step after every epoch. The outcomes of the  train_one_epoch  and the  evaluate  functions are collected and parsed such that a report can be generated. 

 
 
 
  
 
 
 
 
 In [ ]: 
 
      
     params   =   [  p   for   p   in   model  .  parameters  ()   if   p  .  requires_grad  ] 
 optimizer   =   torch  .  optim  .  SGD  (  params  ,   lr  =  0.005  ,   momentum  =  0.9  ,   weight_decay  =  0.0005  ) 
 scheduler   =   torch  .  optim  .  lr_scheduler  .  StepLR  (  optimizer  ,   step_size  =  3  ) 
 device   =   torch  .  device  (  "cuda"  )   if   torch  .  cuda  .  is_available  ()   else   torch  .  device  (  "cpu"  ) 
 model  .  to  (  device  ) 
 freq   =   math  .  ceil  (  len  (  train_dataset  )   /   batch_size   /   4  ) 
 report  ,   early_stop_metrics   =   [],   [] 
 epoch   =   0 
 while   True  : 
     epoch   +=   1 
     train_result   =   train_one_epoch  (  model  ,   optimizer  ,   train_loader  ,   device  ,   epoch  ,   freq  ) 
     train_metrics   =   train_result  .  __str__  () 
     eval_result   =   evaluate  (  model  ,   validate_loader  ,   device  =  device  ) 
     buffer   =   io  .  StringIO  () 
     with   contextlib  .  redirect_stdout  (  buffer  ):   # Little trick to capture eval_result.summarize() into a buffer. 
         eval_result  .  summarize  () 
     eval_metrics   =   buffer  .  getvalue  () 
     record   =   parse_pycoco_metrics  (  epoch  ,   train_metrics  ,   eval_metrics  ) 
     report  .  append  (  record  ) 
     scheduler  .  step  () 
     early_stop_metric   =   record  [  "Average Precision @.50IoU "  ] 
     early_stop_metrics  .  append  (  early_stop_metric  ) 
     if   len  (  early_stop_metrics  )   >=   5   and   early_stop_metric   <=   statistics  .  mean  (  early_stop_metrics  [  -  5  :]): 
         print  (  "Early stopped at epoch"  ,   epoch  ,   "result:"  ,   early_stop_metric  ,   "<= mean("  ,   early_stop_metrics  [  -  5  :],   ")"  ) 
         break 
  

      
 
 
 

 
 
 


 
 
    
      


 
 Epoch: [1]  [  0/502]  eta: 0:20:24  lr: 0.005000  loss: 4.6317 (4.6317)  loss_classifier: 4.1095 (4.1095)  loss_box_reg: 0.2753 (0.2753)  loss_objectness: 0.2338 (0.2338)  loss_rpn_box_reg: 0.0130 (0.0130)  time: 2.4391  data: 0.3108  max mem: 10665
Epoch: [1]  [126/502]  eta: 0:05:34  lr: 0.005000  loss: 1.0794 (1.1265)  loss_classifier: 0.6237 (0.6931)  loss_box_reg: 0.4276 (0.3909)  loss_objectness: 0.0116 (0.0342)  loss_rpn_box_reg: 0.0052 (0.0083)  time: 0.8688  data: 0.2756  max mem: 18264
Epoch: [1]  [252/502]  eta: 0:03:42  lr: 0.005000  loss: 0.7889 (1.0559)  loss_classifier: 0.5103 (0.6433)  loss_box_reg: 0.2896 (0.3808)  loss_objectness: 0.0099 (0.0242)  loss_rpn_box_reg: 0.0052 (0.0076)  time: 0.8888  data: 0.2831  max mem: 18264
Epoch: [1]  [378/502]  eta: 0:01:50  lr: 0.005000  loss: 0.7503 (0.9685)  loss_classifier: 0.4973 (0.6005)  loss_box_reg: 0.2415 (0.3405)  loss_objectness: 0.0086 (0.0205)  loss_rpn_box_reg: 0.0052 (0.0071)  time: 0.8766  data: 0.2709  max mem: 18264
Epoch: [1]  [501/502]  eta: 0:00:00  lr: 0.005000  loss: 0.5974 (0.8907)  loss_classifier: 0.3871 (0.5589)  loss_box_reg: 0.1893 (0.3076)  loss_objectness: 0.0075 (0.0176)  loss_rpn_box_reg: 0.0041 (0.0065)  time: 0.8950  data: 0.2733  max mem: 18264
Epoch: [1] Total time: 0:07:24 (0.8864 s / it)
creating index...
index created!
Test:  [  0/642]  eta: 0:00:45  model_time: 0.0430 (0.0430)  evaluator_time: 0.0048 (0.0048)  time: 0.0707  data: 0.0169  max mem: 18264
Test:  [100/642]  eta: 0:00:32  model_time: 0.0384 (0.0388)  evaluator_time: 0.0016 (0.0019)  time: 0.0598  data: 0.0151  max mem: 18264
Test:  [200/642]  eta: 0:00:26  model_time: 0.0377 (0.0387)  evaluator_time: 0.0015 (0.0023)  time: 0.0594  data: 0.0155  max mem: 18264
Test:  [300/642]  eta: 0:00:20  model_time: 0.0388 (0.0389)  evaluator_time: 0.0017 (0.0022)  time: 0.0593  data: 0.0145  max mem: 18264
Test:  [400/642]  eta: 0:00:14  model_time: 0.0391 (0.0390)  evaluator_time: 0.0017 (0.0021)  time: 0.0603  data: 0.0148  max mem: 18264
Test:  [500/642]  eta: 0:00:08  model_time: 0.0386 (0.0391)  evaluator_time: 0.0014 (0.0021)  time: 0.0591  data: 0.0143  max mem: 18264
Test:  [600/642]  eta: 0:00:02  model_time: 0.0384 (0.0392)  evaluator_time: 0.0017 (0.0021)  time: 0.0623  data: 0.0169  max mem: 18264
Test:  [641/642]  eta: 0:00:00  model_time: 0.0398 (0.0392)  evaluator_time: 0.0019 (0.0021)  time: 0.0624  data: 0.0165  max mem: 18264
Test: Total time: 0:00:38 (0.0604 s / it)
Averaged stats: model_time: 0.0398 (0.0392)  evaluator_time: 0.0019 (0.0021)
Accumulating evaluation results...
DONE (t=0.37s).
IoU metric: bbox
 Average Precision  (AP) @[ IoU=0.50:0.95 | area=   all | maxDets=100 ] = 0.189
 Average Precision  (AP) @[ IoU=0.50      | area=   all | maxDets=100 ] = 0.311
 Average Precision  (AP) @[ IoU=0.75      | area=   all | maxDets=100 ] = 0.214
 Average Precision  (AP) @[ IoU=0.50:0.95 | area= small | maxDets=100 ] = -1.000
 Average Precision  (AP) @[ IoU=0.50:0.95 | area=medium | maxDets=100 ] = 0.088
 Average Precision  (AP) @[ IoU=0.50:0.95 | area= large | maxDets=100 ] = 0.201
 Average Recall     (AR) @[ IoU=0.50:0.95 | area=   all | maxDets=  1 ] = 0.143
 Average Recall     (AR) @[ IoU=0.50:0.95 | area=   all | maxDets= 10 ] = 0.400
 Average Recall     (AR) @[ IoU=0.50:0.95 | area=   all | maxDets=100 ] = 0.419
 Average Recall     (AR) @[ IoU=0.50:0.95 | area= small | maxDets=100 ] = -1.000
 Average Recall     (AR) @[ IoU=0.50:0.95 | area=medium | maxDets=100 ] = 0.178
 Average Recall     (AR) @[ IoU=0.50:0.95 | area= large | maxDets=100 ] = 0.436
Epoch: [2]  [  0/502]  eta: 0:06:18  lr: 0.005000  loss: 0.9898 (0.9898)  loss_classifier: 0.6885 (0.6885)  loss_box_reg: 0.2813 (0.2813)  loss_objectness: 0.0098 (0.0098)  loss_rpn_box_reg: 0.0102 (0.0102)  time: 0.7533  data: 0.2277  max mem: 18264
Epoch: [2]  [126/502]  eta: 0:05:09  lr: 0.005000  loss: 0.4848 (0.5858)  loss_classifier: 0.3202 (0.3800)  loss_box_reg: 0.1754 (0.1920)  loss_objectness: 0.0045 (0.0086)  loss_rpn_box_reg: 0.0031 (0.0051)  time: 0.8105  data: 0.1851  max mem: 18266
Epoch: [2]  [252/502]  eta: 0:03:25  lr: 0.005000  loss: 0.4997 (0.5811)  loss_classifier: 0.3279 (0.3707)  loss_box_reg: 0.1926 (0.1966)  loss_objectness: 0.0040 (0.0087)  loss_rpn_box_reg: 0.0034 (0.0051)  time: 0.8120  data: 0.2060  max mem: 19163
Epoch: [2]  [378/502]  eta: 0:01:42  lr: 0.005000  loss: 0.4943 (0.5607)  loss_classifier: 0.3275 (0.3532)  loss_box_reg: 0.1773 (0.1941)  loss_objectness: 0.0070 (0.0084)  loss_rpn_box_reg: 0.0051 (0.0050)  time: 0.8345  data: 0.2109  max mem: 19163
Epoch: [2]  [501/502]  eta: 0:00:00  lr: 0.005000  loss: 0.3950 (0.5456)  loss_classifier: 0.2188 (0.3389)  loss_box_reg: 0.1660 (0.1933)  loss_objectness: 0.0042 (0.0084)  loss_rpn_box_reg: 0.0031 (0.0050)  time: 0.8102  data: 0.1849  max mem: 19612
Epoch: [2] Total time: 0:06:53 (0.8236 s / it)
creating index...
index created!
Test:  [  0/642]  eta: 0:00:40  model_time: 0.0409 (0.0409)  evaluator_time: 0.0038 (0.0038)  time: 0.0635  data: 0.0149  max mem: 19612
Test:  [100/642]  eta: 0:00:33  model_time: 0.0394 (0.0401)  evaluator_time: 0.0019 (0.0021)  time: 0.0608  data: 0.0146  max mem: 19612
Test:  [200/642]  eta: 0:00:27  model_time: 0.0387 (0.0403)  evaluator_time: 0.0015 (0.0020)  time: 0.0597  data: 0.0153  max mem: 19612
Test:  [300/642]  eta: 0:00:20  model_time: 0.0401 (0.0404)  evaluator_time: 0.0013 (0.0019)  time: 0.0597  data: 0.0143  max mem: 19612
Test:  [400/642]  eta: 0:00:14  model_time: 0.0401 (0.0404)  evaluator_time: 0.0016 (0.0019)  time: 0.0607  data: 0.0140  max mem: 19612
Test:  [500/642]  eta: 0:00:08  model_time: 0.0400 (0.0404)  evaluator_time: 0.0017 (0.0020)  time: 0.0599  data: 0.0140  max mem: 19612
Test:  [600/642]  eta: 0:00:02  model_time: 0.0394 (0.0405)  evaluator_time: 0.0018 (0.0021)  time: 0.0638  data: 0.0166  max mem: 19612
Test:  [641/642]  eta: 0:00:00  model_time: 0.0406 (0.0405)  evaluator_time: 0.0018 (0.0021)  time: 0.0623  data: 0.0154  max mem: 19612
Test: Total time: 0:00:39 (0.0613 s / it)
Averaged stats: model_time: 0.0406 (0.0405)  evaluator_time: 0.0018 (0.0021)
Accumulating evaluation results...
DONE (t=0.36s).
IoU metric: bbox
 Average Precision  (AP) @[ IoU=0.50:0.95 | area=   all | maxDets=100 ] = 0.369
 Average Precision  (AP) @[ IoU=0.50      | area=   all | maxDets=100 ] = 0.599
 Average Precision  (AP) @[ IoU=0.75      | area=   all | maxDets=100 ] = 0.431
 Average Precision  (AP) @[ IoU=0.50:0.95 | area= small | maxDets=100 ] = -1.000
 Average Precision  (AP) @[ IoU=0.50:0.95 | area=medium | maxDets=100 ] = 0.247
 Average Precision  (AP) @[ IoU=0.50:0.95 | area= large | maxDets=100 ] = 0.385
 Average Recall     (AR) @[ IoU=0.50:0.95 | area=   all | maxDets=  1 ] = 0.180
 Average Recall     (AR) @[ IoU=0.50:0.95 | area=   all | maxDets= 10 ] = 0.543
 Average Recall     (AR) @[ IoU=0.50:0.95 | area=   all | maxDets=100 ] = 0.595
 Average Recall     (AR) @[ IoU=0.50:0.95 | area= small | maxDets=100 ] = -1.000
 Average Recall     (AR) @[ IoU=0.50:0.95 | area=medium | maxDets=100 ] = 0.433
 Average Recall     (AR) @[ IoU=0.50:0.95 | area= large | maxDets=100 ] = 0.607
Epoch: [3]  [  0/502]  eta: 0:06:06  lr: 0.005000  loss: 0.4379 (0.4379)  loss_classifier: 0.2766 (0.2766)  loss_box_reg: 0.1549 (0.1549)  loss_objectness: 0.0038 (0.0038)  loss_rpn_box_reg: 0.0026 (0.0026)  time: 0.7311  data: 0.1998  max mem: 19612
Epoch: [3]  [126/502]  eta: 0:05:13  lr: 0.005000  loss: 0.4091 (0.4370)  loss_classifier: 0.2534 (0.2550)  loss_box_reg: 0.1667 (0.1722)  loss_objectness: 0.0041 (0.0057)  loss_rpn_box_reg: 0.0033 (0.0041)  time: 0.8459  data: 0.2042  max mem: 19612
Epoch: [3]  [252/502]  eta: 0:03:26  lr: 0.005000  loss: 0.4446 (0.4211)  loss_classifier: 0.2566 (0.2423)  loss_box_reg: 0.1831 (0.1690)  loss_objectness: 0.0048 (0.0056)  loss_rpn_box_reg: 0.0030 (0.0041)  time: 0.8048  data: 0.2007  max mem: 19612
Epoch: [3]  [378/502]  eta: 0:01:43  lr: 0.005000  loss: 0.4381 (0.4125)  loss_classifier: 0.2386 (0.2355)  loss_box_reg: 0.1809 (0.1672)  loss_objectness: 0.0034 (0.0056)  loss_rpn_box_reg: 0.0037 (0.0041)  time: 0.8299  data: 0.2008  max mem: 19612
Epoch: [3]  [501/502]  eta: 0:00:00  lr: 0.005000  loss: 0.3367 (0.4079)  loss_classifier: 0.1693 (0.2295)  loss_box_reg: 0.1494 (0.1683)  loss_objectness: 0.0032 (0.0058)  loss_rpn_box_reg: 0.0022 (0.0043)  time: 0.8494  data: 0.2058  max mem: 19612
Epoch: [3] Total time: 0:06:59 (0.8359 s / it)
creating index...
index created!
Test:  [  0/642]  eta: 0:00:40  model_time: 0.0409 (0.0409)  evaluator_time: 0.0042 (0.0042)  time: 0.0634  data: 0.0143  max mem: 19612
Test:  [100/642]  eta: 0:00:33  model_time: 0.0406 (0.0408)  evaluator_time: 0.0019 (0.0020)  time: 0.0619  data: 0.0150  max mem: 19612
Test:  [200/642]  eta: 0:00:27  model_time: 0.0393 (0.0407)  evaluator_time: 0.0014 (0.0019)  time: 0.0609  data: 0.0157  max mem: 19612
Test:  [300/642]  eta: 0:00:21  model_time: 0.0403 (0.0409)  evaluator_time: 0.0014 (0.0018)  time: 0.0609  data: 0.0146  max mem: 19612
Test:  [400/642]  eta: 0:00:14  model_time: 0.0408 (0.0410)  evaluator_time: 0.0015 (0.0018)  time: 0.0615  data: 0.0145  max mem: 19612
Test:  [500/642]  eta: 0:00:08  model_time: 0.0398 (0.0410)  evaluator_time: 0.0017 (0.0021)  time: 0.0600  data: 0.0145  max mem: 19612
Test:  [600/642]  eta: 0:00:02  model_time: 0.0404 (0.0410)  evaluator_time: 0.0019 (0.0021)  time: 0.0643  data: 0.0163  max mem: 19612
Test:  [641/642]  eta: 0:00:00  model_time: 0.0412 (0.0410)  evaluator_time: 0.0018 (0.0021)  time: 0.0636  data: 0.0153  max mem: 19612
Test: Total time: 0:00:39 (0.0622 s / it)
Averaged stats: model_time: 0.0412 (0.0410)  evaluator_time: 0.0018 (0.0021)
Accumulating evaluation results...
DONE (t=0.33s).
IoU metric: bbox
 Average Precision  (AP) @[ IoU=0.50:0.95 | area=   all | maxDets=100 ] = 0.447
 Average Precision  (AP) @[ IoU=0.50      | area=   all | maxDets=100 ] = 0.714
 Average Precision  (AP) @[ IoU=0.75      | area=   all | maxDets=100 ] = 0.505
 Average Precision  (AP) @[ IoU=0.50:0.95 | area= small | maxDets=100 ] = -1.000
 Average Precision  (AP) @[ IoU=0.50:0.95 | area=medium | maxDets=100 ] = 0.344
 Average Precision  (AP) @[ IoU=0.50:0.95 | area= large | maxDets=100 ] = 0.460
 Average Recall     (AR) @[ IoU=0.50:0.95 | area=   all | maxDets=  1 ] = 0.190
 Average Recall     (AR) @[ IoU=0.50:0.95 | area=   all | maxDets= 10 ] = 0.570
 Average Recall     (AR) @[ IoU=0.50:0.95 | area=   all | maxDets=100 ] = 0.634
 Average Recall     (AR) @[ IoU=0.50:0.95 | area= small | maxDets=100 ] = -1.000
 Average Recall     (AR) @[ IoU=0.50:0.95 | area=medium | maxDets=100 ] = 0.505
 Average Recall     (AR) @[ IoU=0.50:0.95 | area= large | maxDets=100 ] = 0.642
Epoch: [4]  [  0/502]  eta: 0:07:46  lr: 0.000500  loss: 0.3749 (0.3749)  loss_classifier: 0.1914 (0.1914)  loss_box_reg: 0.1780 (0.1780)  loss_objectness: 0.0010 (0.0010)  loss_rpn_box_reg: 0.0044 (0.0044)  time: 0.9295  data: 0.2512  max mem: 19612
Epoch: [4]  [126/502]  eta: 0:05:12  lr: 0.000500  loss: 0.2398 (0.3056)  loss_classifier: 0.1401 (0.1701)  loss_box_reg: 0.0882 (0.1279)  loss_objectness: 0.0027 (0.0039)  loss_rpn_box_reg: 0.0024 (0.0036)  time: 0.8218  data: 0.1981  max mem: 19612
Epoch: [4]  [252/502]  eta: 0:03:27  lr: 0.000500  loss: 0.2742 (0.2853)  loss_classifier: 0.1458 (0.1581)  loss_box_reg: 0.1260 (0.1199)  loss_objectness: 0.0031 (0.0038)  loss_rpn_box_reg: 0.0031 (0.0035)  time: 0.8164  data: 0.2100  max mem: 19612
Epoch: [4]  [378/502]  eta: 0:01:43  lr: 0.000500  loss: 0.2754 (0.2753)  loss_classifier: 0.1442 (0.1528)  loss_box_reg: 0.1050 (0.1152)  loss_objectness: 0.0030 (0.0038)  loss_rpn_box_reg: 0.0031 (0.0035)  time: 0.8668  data: 0.2268  max mem: 19612
Epoch: [4]  [501/502]  eta: 0:00:00  lr: 0.000500  loss: 0.2816 (0.2735)  loss_classifier: 0.1539 (0.1511)  loss_box_reg: 0.1133 (0.1153)  loss_objectness: 0.0032 (0.0037)  loss_rpn_box_reg: 0.0028 (0.0035)  time: 0.8532  data: 0.2038  max mem: 19612
Epoch: [4] Total time: 0:07:02 (0.8413 s / it)
creating index...
index created!
Test:  [  0/642]  eta: 0:00:41  model_time: 0.0427 (0.0427)  evaluator_time: 0.0040 (0.0040)  time: 0.0650  data: 0.0143  max mem: 19612
Test:  [100/642]  eta: 0:00:33  model_time: 0.0401 (0.0405)  evaluator_time: 0.0015 (0.0018)  time: 0.0611  data: 0.0150  max mem: 19612
Test:  [200/642]  eta: 0:00:27  model_time: 0.0397 (0.0405)  evaluator_time: 0.0013 (0.0017)  time: 0.0606  data: 0.0154  max mem: 19612
Test:  [300/642]  eta: 0:00:21  model_time: 0.0402 (0.0408)  evaluator_time: 0.0013 (0.0017)  time: 0.0607  data: 0.0144  max mem: 19612
Test:  [400/642]  eta: 0:00:14  model_time: 0.0412 (0.0408)  evaluator_time: 0.0015 (0.0017)  time: 0.0615  data: 0.0142  max mem: 19612
Test:  [500/642]  eta: 0:00:08  model_time: 0.0401 (0.0409)  evaluator_time: 0.0015 (0.0017)  time: 0.0602  data: 0.0142  max mem: 19612
Test:  [600/642]  eta: 0:00:02  model_time: 0.0407 (0.0409)  evaluator_time: 0.0016 (0.0018)  time: 0.0652  data: 0.0166  max mem: 19612
Test:  [641/642]  eta: 0:00:00  model_time: 0.0416 (0.0409)  evaluator_time: 0.0018 (0.0018)  time: 0.0636  data: 0.0157  max mem: 19612
Test: Total time: 0:00:39 (0.0617 s / it)
Averaged stats: model_time: 0.0416 (0.0409)  evaluator_time: 0.0018 (0.0018)
Accumulating evaluation results...
DONE (t=0.30s).
IoU metric: bbox
 Average Precision  (AP) @[ IoU=0.50:0.95 | area=   all | maxDets=100 ] = 0.575
 Average Precision  (AP) @[ IoU=0.50      | area=   all | maxDets=100 ] = 0.800
 Average Precision  (AP) @[ IoU=0.75      | area=   all | maxDets=100 ] = 0.672
 Average Precision  (AP) @[ IoU=0.50:0.95 | area= small | maxDets=100 ] = -1.000
 Average Precision  (AP) @[ IoU=0.50:0.95 | area=medium | maxDets=100 ] = 0.407
 Average Precision  (AP) @[ IoU=0.50:0.95 | area= large | maxDets=100 ] = 0.586
 Average Recall     (AR) @[ IoU=0.50:0.95 | area=   all | maxDets=  1 ] = 0.216
 Average Recall     (AR) @[ IoU=0.50:0.95 | area=   all | maxDets= 10 ] = 0.653
 Average Recall     (AR) @[ IoU=0.50:0.95 | area=   all | maxDets=100 ] = 0.724
 Average Recall     (AR) @[ IoU=0.50:0.95 | area= small | maxDets=100 ] = -1.000
 Average Recall     (AR) @[ IoU=0.50:0.95 | area=medium | maxDets=100 ] = 0.539
 Average Recall     (AR) @[ IoU=0.50:0.95 | area= large | maxDets=100 ] = 0.735
Epoch: [5]  [  0/502]  eta: 0:06:55  lr: 0.000500  loss: 0.3472 (0.3472)  loss_classifier: 0.2040 (0.2040)  loss_box_reg: 0.1395 (0.1395)  loss_objectness: 0.0020 (0.0020)  loss_rpn_box_reg: 0.0017 (0.0017)  time: 0.8286  data: 0.2136  max mem: 19612
Epoch: [5]  [126/502]  eta: 0:05:20  lr: 0.000500  loss: 0.2068 (0.2495)  loss_classifier: 0.1267 (0.1388)  loss_box_reg: 0.0931 (0.1044)  loss_objectness: 0.0023 (0.0031)  loss_rpn_box_reg: 0.0025 (0.0032)  time: 0.8358  data: 0.2058  max mem: 19612
Epoch: [5]  [252/502]  eta: 0:03:31  lr: 0.000500  loss: 0.2311 (0.2480)  loss_classifier: 0.1206 (0.1342)  loss_box_reg: 0.0998 (0.1072)  loss_objectness: 0.0026 (0.0033)  loss_rpn_box_reg: 0.0025 (0.0033)  time: 0.8488  data: 0.2331  max mem: 19612
Epoch: [5]  [378/502]  eta: 0:01:44  lr: 0.000500  loss: 0.2147 (0.2432)  loss_classifier: 0.1138 (0.1309)  loss_box_reg: 0.0886 (0.1059)  loss_objectness: 0.0016 (0.0031)  loss_rpn_box_reg: 0.0028 (0.0033)  time: 0.8516  data: 0.2142  max mem: 19612
Epoch: [5]  [501/502]  eta: 0:00:00  lr: 0.000500  loss: 0.1983 (0.2414)  loss_classifier: 0.1137 (0.1299)  loss_box_reg: 0.0858 (0.1051)  loss_objectness: 0.0010 (0.0031)  loss_rpn_box_reg: 0.0017 (0.0033)  time: 0.8465  data: 0.2119  max mem: 19612
Epoch: [5] Total time: 0:07:03 (0.8441 s / it)
creating index...
index created!
Test:  [  0/642]  eta: 0:00:40  model_time: 0.0410 (0.0410)  evaluator_time: 0.0040 (0.0040)  time: 0.0631  data: 0.0141  max mem: 19612
Test:  [100/642]  eta: 0:00:33  model_time: 0.0403 (0.0405)  evaluator_time: 0.0015 (0.0018)  time: 0.0612  data: 0.0149  max mem: 19612
Test:  [200/642]  eta: 0:00:27  model_time: 0.0401 (0.0406)  evaluator_time: 0.0012 (0.0017)  time: 0.0613  data: 0.0157  max mem: 19612
Test:  [300/642]  eta: 0:00:21  model_time: 0.0400 (0.0407)  evaluator_time: 0.0011 (0.0020)  time: 0.0602  data: 0.0146  max mem: 19612
Test:  [400/642]  eta: 0:00:14  model_time: 0.0408 (0.0407)  evaluator_time: 0.0013 (0.0019)  time: 0.0611  data: 0.0143  max mem: 19612
Test:  [500/642]  eta: 0:00:08  model_time: 0.0401 (0.0408)  evaluator_time: 0.0014 (0.0019)  time: 0.0606  data: 0.0142  max mem: 19612
Test:  [600/642]  eta: 0:00:02  model_time: 0.0403 (0.0408)  evaluator_time: 0.0016 (0.0019)  time: 0.0648  data: 0.0162  max mem: 19612
Test:  [641/642]  eta: 0:00:00  model_time: 0.0412 (0.0409)  evaluator_time: 0.0017 (0.0019)  time: 0.0634  data: 0.0157  max mem: 19612
Test: Total time: 0:00:39 (0.0618 s / it)
Averaged stats: model_time: 0.0412 (0.0409)  evaluator_time: 0.0017 (0.0019)
Accumulating evaluation results...
DONE (t=0.29s).
IoU metric: bbox
 Average Precision  (AP) @[ IoU=0.50:0.95 | area=   all | maxDets=100 ] = 0.592
 Average Precision  (AP) @[ IoU=0.50      | area=   all | maxDets=100 ] = 0.812
 Average Precision  (AP) @[ IoU=0.75      | area=   all | maxDets=100 ] = 0.699
 Average Precision  (AP) @[ IoU=0.50:0.95 | area= small | maxDets=100 ] = -1.000
 Average Precision  (AP) @[ IoU=0.50:0.95 | area=medium | maxDets=100 ] = 0.425
 Average Precision  (AP) @[ IoU=0.50:0.95 | area= large | maxDets=100 ] = 0.603
 Average Recall     (AR) @[ IoU=0.50:0.95 | area=   all | maxDets=  1 ] = 0.217
 Average Recall     (AR) @[ IoU=0.50:0.95 | area=   all | maxDets= 10 ] = 0.658
 Average Recall     (AR) @[ IoU=0.50:0.95 | area=   all | maxDets=100 ] = 0.731
 Average Recall     (AR) @[ IoU=0.50:0.95 | area= small | maxDets=100 ] = -1.000
 Average Recall     (AR) @[ IoU=0.50:0.95 | area=medium | maxDets=100 ] = 0.576
 Average Recall     (AR) @[ IoU=0.50:0.95 | area= large | maxDets=100 ] = 0.742
Epoch: [6]  [  0/502]  eta: 0:06:51  lr: 0.000500  loss: 0.1886 (0.1886)  loss_classifier: 0.0974 (0.0974)  loss_box_reg: 0.0879 (0.0879)  loss_objectness: 0.0019 (0.0019)  loss_rpn_box_reg: 0.0014 (0.0014)  time: 0.8198  data: 0.1993  max mem: 19612
Epoch: [6]  [126/502]  eta: 0:05:15  lr: 0.000500  loss: 0.1913 (0.2226)  loss_classifier: 0.0975 (0.1174)  loss_box_reg: 0.0895 (0.0993)  loss_objectness: 0.0017 (0.0028)  loss_rpn_box_reg: 0.0024 (0.0031)  time: 0.8512  data: 0.2289  max mem: 19612
Epoch: [6]  [252/502]  eta: 0:03:30  lr: 0.000500  loss: 0.2156 (0.2223)  loss_classifier: 0.1188 (0.1172)  loss_box_reg: 0.0914 (0.0991)  loss_objectness: 0.0017 (0.0029)  loss_rpn_box_reg: 0.0026 (0.0032)  time: 0.8298  data: 0.2038  max mem: 19612
Epoch: [6]  [378/502]  eta: 0:01:44  lr: 0.000500  loss: 0.2076 (0.2249)  loss_classifier: 0.1120 (0.1176)  loss_box_reg: 0.0913 (0.1011)  loss_objectness: 0.0028 (0.0029)  loss_rpn_box_reg: 0.0024 (0.0033)  time: 0.8353  data: 0.2118  max mem: 19612
Epoch: [6]  [501/502]  eta: 0:00:00  lr: 0.000500  loss: 0.2116 (0.2234)  loss_classifier: 0.1058 (0.1169)  loss_box_reg: 0.1062 (0.1006)  loss_objectness: 0.0019 (0.0028)  loss_rpn_box_reg: 0.0030 (0.0032)  time: 0.8219  data: 0.2056  max mem: 19612
Epoch: [6] Total time: 0:07:02 (0.8421 s / it)
creating index...
index created!
Test:  [  0/642]  eta: 0:00:41  model_time: 0.0426 (0.0426)  evaluator_time: 0.0038 (0.0038)  time: 0.0653  data: 0.0149  max mem: 19612
Test:  [100/642]  eta: 0:00:33  model_time: 0.0409 (0.0407)  evaluator_time: 0.0014 (0.0017)  time: 0.0616  data: 0.0149  max mem: 19612
Test:  [200/642]  eta: 0:00:27  model_time: 0.0390 (0.0405)  evaluator_time: 0.0011 (0.0017)  time: 0.0604  data: 0.0154  max mem: 19612
Test:  [300/642]  eta: 0:00:20  model_time: 0.0403 (0.0407)  evaluator_time: 0.0011 (0.0016)  time: 0.0598  data: 0.0145  max mem: 19612
Test:  [400/642]  eta: 0:00:14  model_time: 0.0397 (0.0408)  evaluator_time: 0.0013 (0.0016)  time: 0.0603  data: 0.0142  max mem: 19612
Test:  [500/642]  eta: 0:00:08  model_time: 0.0394 (0.0407)  evaluator_time: 0.0013 (0.0016)  time: 0.0594  data: 0.0141  max mem: 19612
Test:  [600/642]  eta: 0:00:02  model_time: 0.0386 (0.0407)  evaluator_time: 0.0015 (0.0017)  time: 0.0632  data: 0.0162  max mem: 19612
Test:  [641/642]  eta: 0:00:00  model_time: 0.0403 (0.0407)  evaluator_time: 0.0016 (0.0017)  time: 0.0632  data: 0.0160  max mem: 19612
Test: Total time: 0:00:39 (0.0613 s / it)
Averaged stats: model_time: 0.0403 (0.0407)  evaluator_time: 0.0016 (0.0017)
Accumulating evaluation results...
DONE (t=0.28s).
IoU metric: bbox
 Average Precision  (AP) @[ IoU=0.50:0.95 | area=   all | maxDets=100 ] = 0.601
 Average Precision  (AP) @[ IoU=0.50      | area=   all | maxDets=100 ] = 0.820
 Average Precision  (AP) @[ IoU=0.75      | area=   all | maxDets=100 ] = 0.702
 Average Precision  (AP) @[ IoU=0.50:0.95 | area= small | maxDets=100 ] = -1.000
 Average Precision  (AP) @[ IoU=0.50:0.95 | area=medium | maxDets=100 ] = 0.439
 Average Precision  (AP) @[ IoU=0.50:0.95 | area= large | maxDets=100 ] = 0.613
 Average Recall     (AR) @[ IoU=0.50:0.95 | area=   all | maxDets=  1 ] = 0.219
 Average Recall     (AR) @[ IoU=0.50:0.95 | area=   all | maxDets= 10 ] = 0.662
 Average Recall     (AR) @[ IoU=0.50:0.95 | area=   all | maxDets=100 ] = 0.734
 Average Recall     (AR) @[ IoU=0.50:0.95 | area= small | maxDets=100 ] = -1.000
 Average Recall     (AR) @[ IoU=0.50:0.95 | area=medium | maxDets=100 ] = 0.562
 Average Recall     (AR) @[ IoU=0.50:0.95 | area= large | maxDets=100 ] = 0.745
Epoch: [7]  [  0/502]  eta: 0:06:34  lr: 0.000050  loss: 0.2546 (0.2546)  loss_classifier: 0.1177 (0.1177)  loss_box_reg: 0.1293 (0.1293)  loss_objectness: 0.0035 (0.0035)  loss_rpn_box_reg: 0.0041 (0.0041)  time: 0.7858  data: 0.2017  max mem: 19612
Epoch: [7]  [126/502]  eta: 0:05:18  lr: 0.000050  loss: 0.2194 (0.2232)  loss_classifier: 0.1117 (0.1164)  loss_box_reg: 0.1019 (0.1009)  loss_objectness: 0.0018 (0.0028)  loss_rpn_box_reg: 0.0021 (0.0032)  time: 0.8564  data: 0.2028  max mem: 19612
Epoch: [7]  [252/502]  eta: 0:03:31  lr: 0.000050  loss: 0.2379 (0.2131)  loss_classifier: 0.1228 (0.1107)  loss_box_reg: 0.0997 (0.0967)  loss_objectness: 0.0028 (0.0027)  loss_rpn_box_reg: 0.0024 (0.0030)  time: 0.8600  data: 0.2137  max mem: 19612
Epoch: [7]  [378/502]  eta: 0:01:45  lr: 0.000050  loss: 0.1993 (0.2123)  loss_classifier: 0.1083 (0.1105)  loss_box_reg: 0.0859 (0.0961)  loss_objectness: 0.0014 (0.0027)  loss_rpn_box_reg: 0.0020 (0.0031)  time: 0.8372  data: 0.2126  max mem: 19612
Epoch: [7]  [501/502]  eta: 0:00:00  lr: 0.000050  loss: 0.1934 (0.2101)  loss_classifier: 0.1037 (0.1093)  loss_box_reg: 0.0821 (0.0950)  loss_objectness: 0.0017 (0.0026)  loss_rpn_box_reg: 0.0022 (0.0031)  time: 0.8503  data: 0.2076  max mem: 19612
Epoch: [7] Total time: 0:07:03 (0.8444 s / it)
creating index...
index created!
Test:  [  0/642]  eta: 0:00:41  model_time: 0.0421 (0.0421)  evaluator_time: 0.0040 (0.0040)  time: 0.0647  data: 0.0145  max mem: 19612
Test:  [100/642]  eta: 0:00:33  model_time: 0.0403 (0.0406)  evaluator_time: 0.0014 (0.0017)  time: 0.0614  data: 0.0150  max mem: 19612
Test:  [200/642]  eta: 0:00:27  model_time: 0.0403 (0.0410)  evaluator_time: 0.0012 (0.0016)  time: 0.0611  data: 0.0155  max mem: 19612
Test:  [300/642]  eta: 0:00:21  model_time: 0.0403 (0.0412)  evaluator_time: 0.0011 (0.0016)  time: 0.0601  data: 0.0144  max mem: 19612
Test:  [400/642]  eta: 0:00:14  model_time: 0.0408 (0.0412)  evaluator_time: 0.0013 (0.0016)  time: 0.0612  data: 0.0143  max mem: 19612
Test:  [500/642]  eta: 0:00:08  model_time: 0.0397 (0.0412)  evaluator_time: 0.0013 (0.0016)  time: 0.0602  data: 0.0143  max mem: 19612
Test:  [600/642]  eta: 0:00:02  model_time: 0.0406 (0.0412)  evaluator_time: 0.0015 (0.0017)  time: 0.0646  data: 0.0162  max mem: 19612
Test:  [641/642]  eta: 0:00:00  model_time: 0.0410 (0.0412)  evaluator_time: 0.0016 (0.0017)  time: 0.0635  data: 0.0157  max mem: 19612
Test: Total time: 0:00:39 (0.0618 s / it)
Averaged stats: model_time: 0.0410 (0.0412)  evaluator_time: 0.0016 (0.0017)
Accumulating evaluation results...
DONE (t=0.28s).
IoU metric: bbox
 Average Precision  (AP) @[ IoU=0.50:0.95 | area=   all | maxDets=100 ] = 0.606
 Average Precision  (AP) @[ IoU=0.50      | area=   all | maxDets=100 ] = 0.820
 Average Precision  (AP) @[ IoU=0.75      | area=   all | maxDets=100 ] = 0.708
 Average Precision  (AP) @[ IoU=0.50:0.95 | area= small | maxDets=100 ] = -1.000
 Average Precision  (AP) @[ IoU=0.50:0.95 | area=medium | maxDets=100 ] = 0.428
 Average Precision  (AP) @[ IoU=0.50:0.95 | area= large | maxDets=100 ] = 0.617
 Average Recall     (AR) @[ IoU=0.50:0.95 | area=   all | maxDets=  1 ] = 0.221
 Average Recall     (AR) @[ IoU=0.50:0.95 | area=   all | maxDets= 10 ] = 0.665
 Average Recall     (AR) @[ IoU=0.50:0.95 | area=   all | maxDets=100 ] = 0.737
 Average Recall     (AR) @[ IoU=0.50:0.95 | area= small | maxDets=100 ] = -1.000
 Average Recall     (AR) @[ IoU=0.50:0.95 | area=medium | maxDets=100 ] = 0.557
 Average Recall     (AR) @[ IoU=0.50:0.95 | area= large | maxDets=100 ] = 0.748
Epoch: [8]  [  0/502]  eta: 0:06:12  lr: 0.000050  loss: 0.1603 (0.1603)  loss_classifier: 0.0817 (0.0817)  loss_box_reg: 0.0767 (0.0767)  loss_objectness: 0.0006 (0.0006)  loss_rpn_box_reg: 0.0012 (0.0012)  time: 0.7413  data: 0.2051  max mem: 19612
Epoch: [8]  [126/502]  eta: 0:05:08  lr: 0.000050  loss: 0.1784 (0.2046)  loss_classifier: 0.0883 (0.1042)  loss_box_reg: 0.0821 (0.0943)  loss_objectness: 0.0017 (0.0030)  loss_rpn_box_reg: 0.0020 (0.0031)  time: 0.8008  data: 0.2002  max mem: 19612
Epoch: [8]  [252/502]  eta: 0:03:29  lr: 0.000050  loss: 0.1939 (0.2038)  loss_classifier: 0.1075 (0.1045)  loss_box_reg: 0.0915 (0.0934)  loss_objectness: 0.0017 (0.0029)  loss_rpn_box_reg: 0.0019 (0.0030)  time: 0.8161  data: 0.2214  max mem: 19612
Epoch: [8]  [378/502]  eta: 0:01:44  lr: 0.000050  loss: 0.1834 (0.2083)  loss_classifier: 0.1009 (0.1073)  loss_box_reg: 0.0891 (0.0950)  loss_objectness: 0.0023 (0.0028)  loss_rpn_box_reg: 0.0024 (0.0031)  time: 0.8188  data: 0.2159  max mem: 19612
Epoch: [8]  [501/502]  eta: 0:00:00  lr: 0.000050  loss: 0.1827 (0.2062)  loss_classifier: 0.0865 (0.1064)  loss_box_reg: 0.0883 (0.0941)  loss_objectness: 0.0016 (0.0026)  loss_rpn_box_reg: 0.0022 (0.0031)  time: 0.8171  data: 0.2035  max mem: 19612
Epoch: [8] Total time: 0:07:03 (0.8436 s / it)
creating index...
index created!
Test:  [  0/642]  eta: 0:00:40  model_time: 0.0407 (0.0407)  evaluator_time: 0.0040 (0.0040)  time: 0.0630  data: 0.0143  max mem: 19612
Test:  [100/642]  eta: 0:00:33  model_time: 0.0399 (0.0407)  evaluator_time: 0.0014 (0.0017)  time: 0.0614  data: 0.0149  max mem: 19612
Test:  [200/642]  eta: 0:00:27  model_time: 0.0392 (0.0405)  evaluator_time: 0.0011 (0.0016)  time: 0.0605  data: 0.0156  max mem: 19612
Test:  [300/642]  eta: 0:00:20  model_time: 0.0405 (0.0407)  evaluator_time: 0.0011 (0.0016)  time: 0.0605  data: 0.0147  max mem: 19612
Test:  [400/642]  eta: 0:00:14  model_time: 0.0405 (0.0407)  evaluator_time: 0.0013 (0.0016)  time: 0.0607  data: 0.0143  max mem: 19612
Test:  [500/642]  eta: 0:00:08  model_time: 0.0399 (0.0407)  evaluator_time: 0.0013 (0.0016)  time: 0.0600  data: 0.0143  max mem: 19612
Test:  [600/642]  eta: 0:00:02  model_time: 0.0400 (0.0408)  evaluator_time: 0.0015 (0.0017)  time: 0.0642  data: 0.0163  max mem: 19612
Test:  [641/642]  eta: 0:00:00  model_time: 0.0407 (0.0408)  evaluator_time: 0.0016 (0.0017)  time: 0.0630  data: 0.0158  max mem: 19612
Test: Total time: 0:00:39 (0.0615 s / it)
Averaged stats: model_time: 0.0407 (0.0408)  evaluator_time: 0.0016 (0.0017)
Accumulating evaluation results...
DONE (t=0.27s).
IoU metric: bbox
 Average Precision  (AP) @[ IoU=0.50:0.95 | area=   all | maxDets=100 ] = 0.607
 Average Precision  (AP) @[ IoU=0.50      | area=   all | maxDets=100 ] = 0.822
 Average Precision  (AP) @[ IoU=0.75      | area=   all | maxDets=100 ] = 0.707
 Average Precision  (AP) @[ IoU=0.50:0.95 | area= small | maxDets=100 ] = -1.000
 Average Precision  (AP) @[ IoU=0.50:0.95 | area=medium | maxDets=100 ] = 0.442
 Average Precision  (AP) @[ IoU=0.50:0.95 | area= large | maxDets=100 ] = 0.618
 Average Recall     (AR) @[ IoU=0.50:0.95 | area=   all | maxDets=  1 ] = 0.220
 Average Recall     (AR) @[ IoU=0.50:0.95 | area=   all | maxDets= 10 ] = 0.667
 Average Recall     (AR) @[ IoU=0.50:0.95 | area=   all | maxDets=100 ] = 0.735
 Average Recall     (AR) @[ IoU=0.50:0.95 | area= small | maxDets=100 ] = -1.000
 Average Recall     (AR) @[ IoU=0.50:0.95 | area=medium | maxDets=100 ] = 0.557
 Average Recall     (AR) @[ IoU=0.50:0.95 | area= large | maxDets=100 ] = 0.745
Epoch: [9]  [  0/502]  eta: 0:06:47  lr: 0.000050  loss: 0.2362 (0.2362)  loss_classifier: 0.1334 (0.1334)  loss_box_reg: 0.0992 (0.0992)  loss_objectness: 0.0022 (0.0022)  loss_rpn_box_reg: 0.0014 (0.0014)  time: 0.8124  data: 0.2273  max mem: 19612
Epoch: [9]  [126/502]  eta: 0:05:13  lr: 0.000050  loss: 0.1746 (0.1990)  loss_classifier: 0.0860 (0.1035)  loss_box_reg: 0.0894 (0.0903)  loss_objectness: 0.0015 (0.0024)  loss_rpn_box_reg: 0.0025 (0.0028)  time: 0.8416  data: 0.2106  max mem: 19612
Epoch: [9]  [252/502]  eta: 0:03:28  lr: 0.000050  loss: 0.2365 (0.2040)  loss_classifier: 0.1139 (0.1071)  loss_box_reg: 0.1072 (0.0913)  loss_objectness: 0.0029 (0.0026)  loss_rpn_box_reg: 0.0026 (0.0029)  time: 0.8453  data: 0.2090  max mem: 19612
Epoch: [9]  [378/502]  eta: 0:01:44  lr: 0.000050  loss: 0.1630 (0.2042)  loss_classifier: 0.0823 (0.1063)  loss_box_reg: 0.0826 (0.0924)  loss_objectness: 0.0012 (0.0026)  loss_rpn_box_reg: 0.0015 (0.0030)  time: 0.8724  data: 0.2110  max mem: 19612
Epoch: [9]  [501/502]  eta: 0:00:00  lr: 0.000050  loss: 0.1904 (0.2062)  loss_classifier: 0.1025 (0.1070)  loss_box_reg: 0.0936 (0.0936)  loss_objectness: 0.0028 (0.0026)  loss_rpn_box_reg: 0.0023 (0.0030)  time: 0.8471  data: 0.2077  max mem: 19627
Epoch: [9] Total time: 0:07:02 (0.8412 s / it)
creating index...
index created!
Test:  [  0/642]  eta: 0:00:41  model_time: 0.0413 (0.0413)  evaluator_time: 0.0041 (0.0041)  time: 0.0650  data: 0.0156  max mem: 19627
Test:  [100/642]  eta: 0:00:34  model_time: 0.0414 (0.0409)  evaluator_time: 0.0014 (0.0026)  time: 0.0623  data: 0.0153  max mem: 19627
Test:  [200/642]  eta: 0:00:27  model_time: 0.0394 (0.0409)  evaluator_time: 0.0011 (0.0021)  time: 0.0608  data: 0.0155  max mem: 19627
Test:  [300/642]  eta: 0:00:21  model_time: 0.0411 (0.0411)  evaluator_time: 0.0012 (0.0019)  time: 0.0611  data: 0.0145  max mem: 19627
Test:  [400/642]  eta: 0:00:15  model_time: 0.0407 (0.0411)  evaluator_time: 0.0014 (0.0018)  time: 0.0615  data: 0.0145  max mem: 19627
Test:  [500/642]  eta: 0:00:08  model_time: 0.0402 (0.0412)  evaluator_time: 0.0012 (0.0018)  time: 0.0606  data: 0.0143  max mem: 19627
Test:  [600/642]  eta: 0:00:02  model_time: 0.0402 (0.0411)  evaluator_time: 0.0016 (0.0019)  time: 0.0641  data: 0.0164  max mem: 19627
Test:  [641/642]  eta: 0:00:00  model_time: 0.0414 (0.0411)  evaluator_time: 0.0017 (0.0019)  time: 0.0637  data: 0.0159  max mem: 19627
Test: Total time: 0:00:39 (0.0621 s / it)
Averaged stats: model_time: 0.0414 (0.0411)  evaluator_time: 0.0017 (0.0019)
Accumulating evaluation results...
DONE (t=0.28s).
IoU metric: bbox
 Average Precision  (AP) @[ IoU=0.50:0.95 | area=   all | maxDets=100 ] = 0.606
 Average Precision  (AP) @[ IoU=0.50      | area=   all | maxDets=100 ] = 0.821
 Average Precision  (AP) @[ IoU=0.75      | area=   all | maxDets=100 ] = 0.705
 Average Precision  (AP) @[ IoU=0.50:0.95 | area= small | maxDets=100 ] = -1.000
 Average Precision  (AP) @[ IoU=0.50:0.95 | area=medium | maxDets=100 ] = 0.450
 Average Precision  (AP) @[ IoU=0.50:0.95 | area= large | maxDets=100 ] = 0.618
 Average Recall     (AR) @[ IoU=0.50:0.95 | area=   all | maxDets=  1 ] = 0.221
 Average Recall     (AR) @[ IoU=0.50:0.95 | area=   all | maxDets= 10 ] = 0.665
 Average Recall     (AR) @[ IoU=0.50:0.95 | area=   all | maxDets=100 ] = 0.736
 Average Recall     (AR) @[ IoU=0.50:0.95 | area= small | maxDets=100 ] = -1.000
 Average Recall     (AR) @[ IoU=0.50:0.95 | area=medium | maxDets=100 ] = 0.572
 Average Recall     (AR) @[ IoU=0.50:0.95 | area= large | maxDets=100 ] = 0.744
Epoch: [10]  [  0/502]  eta: 0:06:34  lr: 0.000005  loss: 0.1364 (0.1364)  loss_classifier: 0.0641 (0.0641)  loss_box_reg: 0.0685 (0.0685)  loss_objectness: 0.0020 (0.0020)  loss_rpn_box_reg: 0.0017 (0.0017)  time: 0.7860  data: 0.2201  max mem: 19627
Epoch: [10]  [126/502]  eta: 0:05:18  lr: 0.000005  loss: 0.1929 (0.2050)  loss_classifier: 0.0966 (0.1067)  loss_box_reg: 0.0864 (0.0925)  loss_objectness: 0.0020 (0.0028)  loss_rpn_box_reg: 0.0025 (0.0030)  time: 0.8425  data: 0.2153  max mem: 19627
Epoch: [10]  [252/502]  eta: 0:03:30  lr: 0.000005  loss: 0.2043 (0.2036)  loss_classifier: 0.0972 (0.1045)  loss_box_reg: 0.0917 (0.0936)  loss_objectness: 0.0011 (0.0024)  loss_rpn_box_reg: 0.0027 (0.0030)  time: 0.8785  data: 0.2183  max mem: 19627
Epoch: [10]  [378/502]  eta: 0:01:44  lr: 0.000005  loss: 0.2479 (0.2051)  loss_classifier: 0.1181 (0.1056)  loss_box_reg: 0.1033 (0.0940)  loss_objectness: 0.0016 (0.0024)  loss_rpn_box_reg: 0.0024 (0.0031)  time: 0.8814  data: 0.2354  max mem: 19627
Epoch: [10]  [501/502]  eta: 0:00:00  lr: 0.000005  loss: 0.2174 (0.2033)  loss_classifier: 0.1024 (0.1051)  loss_box_reg: 0.0991 (0.0928)  loss_objectness: 0.0009 (0.0024)  loss_rpn_box_reg: 0.0028 (0.0030)  time: 0.8608  data: 0.2052  max mem: 19627
Epoch: [10] Total time: 0:07:04 (0.8456 s / it)
creating index...
index created!
Test:  [  0/642]  eta: 0:00:40  model_time: 0.0412 (0.0412)  evaluator_time: 0.0039 (0.0039)  time: 0.0637  data: 0.0146  max mem: 19627
Test:  [100/642]  eta: 0:00:33  model_time: 0.0405 (0.0408)  evaluator_time: 0.0014 (0.0017)  time: 0.0614  data: 0.0149  max mem: 19627
Test:  [200/642]  eta: 0:00:27  model_time: 0.0399 (0.0407)  evaluator_time: 0.0011 (0.0017)  time: 0.0609  data: 0.0156  max mem: 19627
Test:  [300/642]  eta: 0:00:21  model_time: 0.0412 (0.0409)  evaluator_time: 0.0012 (0.0016)  time: 0.0613  data: 0.0145  max mem: 19627
Test:  [400/642]  eta: 0:00:14  model_time: 0.0408 (0.0410)  evaluator_time: 0.0014 (0.0016)  time: 0.0614  data: 0.0144  max mem: 19627
Test:  [500/642]  eta: 0:00:08  model_time: 0.0404 (0.0411)  evaluator_time: 0.0013 (0.0017)  time: 0.0608  data: 0.0145  max mem: 19627
Test:  [600/642]  eta: 0:00:02  model_time: 0.0409 (0.0411)  evaluator_time: 0.0015 (0.0017)  time: 0.0648  data: 0.0163  max mem: 19627
Test:  [641/642]  eta: 0:00:00  model_time: 0.0414 (0.0411)  evaluator_time: 0.0017 (0.0017)  time: 0.0640  data: 0.0157  max mem: 19627
Test: Total time: 0:00:39 (0.0620 s / it)
Averaged stats: model_time: 0.0414 (0.0411)  evaluator_time: 0.0017 (0.0017)
Accumulating evaluation results...
DONE (t=0.28s).
IoU metric: bbox
 Average Precision  (AP) @[ IoU=0.50:0.95 | area=   all | maxDets=100 ] = 0.607
 Average Precision  (AP) @[ IoU=0.50      | area=   all | maxDets=100 ] = 0.822
 Average Precision  (AP) @[ IoU=0.75      | area=   all | maxDets=100 ] = 0.706
 Average Precision  (AP) @[ IoU=0.50:0.95 | area= small | maxDets=100 ] = -1.000
 Average Precision  (AP) @[ IoU=0.50:0.95 | area=medium | maxDets=100 ] = 0.438
 Average Precision  (AP) @[ IoU=0.50:0.95 | area= large | maxDets=100 ] = 0.618
 Average Recall     (AR) @[ IoU=0.50:0.95 | area=   all | maxDets=  1 ] = 0.221
 Average Recall     (AR) @[ IoU=0.50:0.95 | area=   all | maxDets= 10 ] = 0.666
 Average Recall     (AR) @[ IoU=0.50:0.95 | area=   all | maxDets=100 ] = 0.737
 Average Recall     (AR) @[ IoU=0.50:0.95 | area= small | maxDets=100 ] = -1.000
 Average Recall     (AR) @[ IoU=0.50:0.95 | area=medium | maxDets=100 ] = 0.556
 Average Recall     (AR) @[ IoU=0.50:0.95 | area= large | maxDets=100 ] = 0.747
Epoch: [11]  [  0/502]  eta: 0:05:39  lr: 0.000005  loss: 0.1661 (0.1661)  loss_classifier: 0.0901 (0.0901)  loss_box_reg: 0.0713 (0.0713)  loss_objectness: 0.0014 (0.0014)  loss_rpn_box_reg: 0.0033 (0.0033)  time: 0.6758  data: 0.0956  max mem: 19627
Epoch: [11]  [126/502]  eta: 0:05:18  lr: 0.000005  loss: 0.1890 (0.2002)  loss_classifier: 0.0933 (0.1023)  loss_box_reg: 0.0898 (0.0927)  loss_objectness: 0.0019 (0.0022)  loss_rpn_box_reg: 0.0030 (0.0030)  time: 0.8665  data: 0.2267  max mem: 19627
Epoch: [11]  [252/502]  eta: 0:03:30  lr: 0.000005  loss: 0.2150 (0.2029)  loss_classifier: 0.1105 (0.1044)  loss_box_reg: 0.0928 (0.0932)  loss_objectness: 0.0018 (0.0024)  loss_rpn_box_reg: 0.0027 (0.0029)  time: 0.8540  data: 0.2118  max mem: 19627
Epoch: [11]  [378/502]  eta: 0:01:44  lr: 0.000005  loss: 0.1949 (0.2029)  loss_classifier: 0.1019 (0.1049)  loss_box_reg: 0.0814 (0.0926)  loss_objectness: 0.0018 (0.0024)  loss_rpn_box_reg: 0.0020 (0.0029)  time: 0.8384  data: 0.2086  max mem: 19627
Epoch: [11]  [501/502]  eta: 0:00:00  lr: 0.000005  loss: 0.1890 (0.2031)  loss_classifier: 0.0931 (0.1049)  loss_box_reg: 0.0890 (0.0927)  loss_objectness: 0.0013 (0.0025)  loss_rpn_box_reg: 0.0023 (0.0030)  time: 0.8412  data: 0.2200  max mem: 19627
Epoch: [11] Total time: 0:07:02 (0.8413 s / it)
creating index...
index created!
Test:  [  0/642]  eta: 0:00:40  model_time: 0.0406 (0.0406)  evaluator_time: 0.0041 (0.0041)  time: 0.0629  data: 0.0141  max mem: 19627
Test:  [100/642]  eta: 0:00:33  model_time: 0.0404 (0.0403)  evaluator_time: 0.0014 (0.0017)  time: 0.0614  data: 0.0150  max mem: 19627
Test:  [200/642]  eta: 0:00:27  model_time: 0.0386 (0.0402)  evaluator_time: 0.0012 (0.0017)  time: 0.0603  data: 0.0157  max mem: 19627
Test:  [300/642]  eta: 0:00:20  model_time: 0.0400 (0.0404)  evaluator_time: 0.0011 (0.0016)  time: 0.0603  data: 0.0147  max mem: 19627
Test:  [400/642]  eta: 0:00:14  model_time: 0.0403 (0.0406)  evaluator_time: 0.0014 (0.0016)  time: 0.0614  data: 0.0145  max mem: 19627
Test:  [500/642]  eta: 0:00:08  model_time: 0.0410 (0.0408)  evaluator_time: 0.0013 (0.0017)  time: 0.0607  data: 0.0144  max mem: 19627
Test:  [600/642]  eta: 0:00:02  model_time: 0.0408 (0.0408)  evaluator_time: 0.0016 (0.0017)  time: 0.0651  data: 0.0169  max mem: 19627
Test:  [641/642]  eta: 0:00:00  model_time: 0.0412 (0.0409)  evaluator_time: 0.0017 (0.0018)  time: 0.0635  data: 0.0158  max mem: 19627
Test: Total time: 0:00:39 (0.0618 s / it)
Averaged stats: model_time: 0.0412 (0.0409)  evaluator_time: 0.0017 (0.0018)
Accumulating evaluation results...
DONE (t=0.28s).
IoU metric: bbox
 Average Precision  (AP) @[ IoU=0.50:0.95 | area=   all | maxDets=100 ] = 0.609
 Average Precision  (AP) @[ IoU=0.50      | area=   all | maxDets=100 ] = 0.822
 Average Precision  (AP) @[ IoU=0.75      | area=   all | maxDets=100 ] = 0.711
 Average Precision  (AP) @[ IoU=0.50:0.95 | area= small | maxDets=100 ] = -1.000
 Average Precision  (AP) @[ IoU=0.50:0.95 | area=medium | maxDets=100 ] = 0.443
 Average Precision  (AP) @[ IoU=0.50:0.95 | area= large | maxDets=100 ] = 0.620
 Average Recall     (AR) @[ IoU=0.50:0.95 | area=   all | maxDets=  1 ] = 0.221
 Average Recall     (AR) @[ IoU=0.50:0.95 | area=   all | maxDets= 10 ] = 0.668
 Average Recall     (AR) @[ IoU=0.50:0.95 | area=   all | maxDets=100 ] = 0.738
 Average Recall     (AR) @[ IoU=0.50:0.95 | area= small | maxDets=100 ] = -1.000
 Average Recall     (AR) @[ IoU=0.50:0.95 | area=medium | maxDets=100 ] = 0.562
 Average Recall     (AR) @[ IoU=0.50:0.95 | area= large | maxDets=100 ] = 0.749
Epoch: [12]  [  0/502]  eta: 0:06:29  lr: 0.000005  loss: 0.1876 (0.1876)  loss_classifier: 0.0824 (0.0824)  loss_box_reg: 0.1019 (0.1019)  loss_objectness: 0.0008 (0.0008)  loss_rpn_box_reg: 0.0026 (0.0026)  time: 0.7767  data: 0.2036  max mem: 19627
Epoch: [12]  [126/502]  eta: 0:05:17  lr: 0.000005  loss: 0.1871 (0.2027)  loss_classifier: 0.0907 (0.1035)  loss_box_reg: 0.0870 (0.0934)  loss_objectness: 0.0019 (0.0027)  loss_rpn_box_reg: 0.0022 (0.0031)  time: 0.8424  data: 0.2127  max mem: 19627
Epoch: [12]  [252/502]  eta: 0:03:30  lr: 0.000005  loss: 0.1989 (0.2033)  loss_classifier: 0.1045 (0.1044)  loss_box_reg: 0.0851 (0.0933)  loss_objectness: 0.0019 (0.0026)  loss_rpn_box_reg: 0.0020 (0.0031)  time: 0.8498  data: 0.2213  max mem: 19627
Epoch: [12]  [378/502]  eta: 0:01:44  lr: 0.000005  loss: 0.1861 (0.2051)  loss_classifier: 0.0903 (0.1061)  loss_box_reg: 0.0874 (0.0932)  loss_objectness: 0.0025 (0.0027)  loss_rpn_box_reg: 0.0024 (0.0030)  time: 0.8054  data: 0.1754  max mem: 19627
Epoch: [12]  [501/502]  eta: 0:00:00  lr: 0.000005  loss: 0.1671 (0.2037)  loss_classifier: 0.0810 (0.1053)  loss_box_reg: 0.0803 (0.0927)  loss_objectness: 0.0010 (0.0026)  loss_rpn_box_reg: 0.0020 (0.0030)  time: 0.8763  data: 0.2126  max mem: 19627
Epoch: [12] Total time: 0:07:02 (0.8420 s / it)
creating index...
index created!
Test:  [  0/642]  eta: 0:00:41  model_time: 0.0427 (0.0427)  evaluator_time: 0.0039 (0.0039)  time: 0.0653  data: 0.0147  max mem: 19627
Test:  [100/642]  eta: 0:00:33  model_time: 0.0411 (0.0410)  evaluator_time: 0.0013 (0.0017)  time: 0.0617  data: 0.0148  max mem: 19627
Test:  [200/642]  eta: 0:00:27  model_time: 0.0400 (0.0411)  evaluator_time: 0.0011 (0.0016)  time: 0.0613  data: 0.0156  max mem: 19627
Test:  [300/642]  eta: 0:00:21  model_time: 0.0408 (0.0414)  evaluator_time: 0.0011 (0.0016)  time: 0.0611  data: 0.0147  max mem: 19627
Test:  [400/642]  eta: 0:00:15  model_time: 0.0409 (0.0413)  evaluator_time: 0.0013 (0.0016)  time: 0.0617  data: 0.0145  max mem: 19627
Test:  [500/642]  eta: 0:00:08  model_time: 0.0401 (0.0413)  evaluator_time: 0.0013 (0.0018)  time: 0.0602  data: 0.0143  max mem: 19627
Test:  [600/642]  eta: 0:00:02  model_time: 0.0398 (0.0413)  evaluator_time: 0.0014 (0.0018)  time: 0.0644  data: 0.0162  max mem: 19627
Test:  [641/642]  eta: 0:00:00  model_time: 0.0406 (0.0413)  evaluator_time: 0.0017 (0.0019)  time: 0.0630  data: 0.0158  max mem: 19627
Test: Total time: 0:00:39 (0.0622 s / it)
Averaged stats: model_time: 0.0406 (0.0413)  evaluator_time: 0.0017 (0.0019)
Accumulating evaluation results...
DONE (t=0.28s).
IoU metric: bbox
 Average Precision  (AP) @[ IoU=0.50:0.95 | area=   all | maxDets=100 ] = 0.611
 Average Precision  (AP) @[ IoU=0.50      | area=   all | maxDets=100 ] = 0.824
 Average Precision  (AP) @[ IoU=0.75      | area=   all | maxDets=100 ] = 0.714
 Average Precision  (AP) @[ IoU=0.50:0.95 | area= small | maxDets=100 ] = -1.000
 Average Precision  (AP) @[ IoU=0.50:0.95 | area=medium | maxDets=100 ] = 0.443
 Average Precision  (AP) @[ IoU=0.50:0.95 | area= large | maxDets=100 ] = 0.621
 Average Recall     (AR) @[ IoU=0.50:0.95 | area=   all | maxDets=  1 ] = 0.220
 Average Recall     (AR) @[ IoU=0.50:0.95 | area=   all | maxDets= 10 ] = 0.669
 Average Recall     (AR) @[ IoU=0.50:0.95 | area=   all | maxDets=100 ] = 0.739
 Average Recall     (AR) @[ IoU=0.50:0.95 | area= small | maxDets=100 ] = -1.000
 Average Recall     (AR) @[ IoU=0.50:0.95 | area=medium | maxDets=100 ] = 0.550
 Average Recall     (AR) @[ IoU=0.50:0.95 | area= large | maxDets=100 ] = 0.751
Epoch: [13]  [  0/502]  eta: 0:08:36  lr: 0.000001  loss: 0.1917 (0.1917)  loss_classifier: 0.0960 (0.0960)  loss_box_reg: 0.0931 (0.0931)  loss_objectness: 0.0008 (0.0008)  loss_rpn_box_reg: 0.0018 (0.0018)  time: 1.0282  data: 0.2191  max mem: 19627
Epoch: [13]  [126/502]  eta: 0:05:18  lr: 0.000001  loss: 0.1726 (0.2014)  loss_classifier: 0.0802 (0.1053)  loss_box_reg: 0.0812 (0.0908)  loss_objectness: 0.0026 (0.0024)  loss_rpn_box_reg: 0.0024 (0.0029)  time: 0.8500  data: 0.2098  max mem: 19627
Epoch: [13]  [252/502]  eta: 0:03:32  lr: 0.000001  loss: 0.2328 (0.2009)  loss_classifier: 0.1034 (0.1036)  loss_box_reg: 0.1149 (0.0920)  loss_objectness: 0.0023 (0.0024)  loss_rpn_box_reg: 0.0038 (0.0029)  time: 0.8225  data: 0.2034  max mem: 19627
Epoch: [13]  [378/502]  eta: 0:01:45  lr: 0.000001  loss: 0.1922 (0.2046)  loss_classifier: 0.0882 (0.1060)  loss_box_reg: 0.0822 (0.0932)  loss_objectness: 0.0014 (0.0024)  loss_rpn_box_reg: 0.0021 (0.0031)  time: 0.8116  data: 0.2129  max mem: 19627
Epoch: [13]  [501/502]  eta: 0:00:00  lr: 0.000001  loss: 0.1510 (0.2032)  loss_classifier: 0.0993 (0.1053)  loss_box_reg: 0.0722 (0.0923)  loss_objectness: 0.0019 (0.0025)  loss_rpn_box_reg: 0.0019 (0.0030)  time: 0.8533  data: 0.2133  max mem: 19627
Epoch: [13] Total time: 0:07:05 (0.8479 s / it)
creating index...
index created!
Test:  [  0/642]  eta: 0:00:42  model_time: 0.0431 (0.0431)  evaluator_time: 0.0040 (0.0040)  time: 0.0658  data: 0.0145  max mem: 19627
Test:  [100/642]  eta: 0:00:33  model_time: 0.0407 (0.0408)  evaluator_time: 0.0014 (0.0017)  time: 0.0618  data: 0.0151  max mem: 19627
Test:  [200/642]  eta: 0:00:27  model_time: 0.0397 (0.0408)  evaluator_time: 0.0012 (0.0017)  time: 0.0607  data: 0.0156  max mem: 19627
Test:  [300/642]  eta: 0:00:21  model_time: 0.0402 (0.0409)  evaluator_time: 0.0012 (0.0016)  time: 0.0606  data: 0.0146  max mem: 19627
Test:  [400/642]  eta: 0:00:14  model_time: 0.0405 (0.0409)  evaluator_time: 0.0013 (0.0016)  time: 0.0613  data: 0.0144  max mem: 19627
Test:  [500/642]  eta: 0:00:08  model_time: 0.0396 (0.0409)  evaluator_time: 0.0014 (0.0017)  time: 0.0599  data: 0.0145  max mem: 19627
Test:  [600/642]  eta: 0:00:02  model_time: 0.0398 (0.0409)  evaluator_time: 0.0014 (0.0017)  time: 0.0643  data: 0.0163  max mem: 19627
Test:  [641/642]  eta: 0:00:00  model_time: 0.0410 (0.0409)  evaluator_time: 0.0017 (0.0018)  time: 0.0634  data: 0.0160  max mem: 19627
Test: Total time: 0:00:39 (0.0618 s / it)
Averaged stats: model_time: 0.0410 (0.0409)  evaluator_time: 0.0017 (0.0018)
Accumulating evaluation results...
DONE (t=0.28s).
IoU metric: bbox
 Average Precision  (AP) @[ IoU=0.50:0.95 | area=   all | maxDets=100 ] = 0.611
 Average Precision  (AP) @[ IoU=0.50      | area=   all | maxDets=100 ] = 0.823
 Average Precision  (AP) @[ IoU=0.75      | area=   all | maxDets=100 ] = 0.714
 Average Precision  (AP) @[ IoU=0.50:0.95 | area= small | maxDets=100 ] = -1.000
 Average Precision  (AP) @[ IoU=0.50:0.95 | area=medium | maxDets=100 ] = 0.452
 Average Precision  (AP) @[ IoU=0.50:0.95 | area= large | maxDets=100 ] = 0.623
 Average Recall     (AR) @[ IoU=0.50:0.95 | area=   all | maxDets=  1 ] = 0.223
 Average Recall     (AR) @[ IoU=0.50:0.95 | area=   all | maxDets= 10 ] = 0.669
 Average Recall     (AR) @[ IoU=0.50:0.95 | area=   all | maxDets=100 ] = 0.737
 Average Recall     (AR) @[ IoU=0.50:0.95 | area= small | maxDets=100 ] = -1.000
 Average Recall     (AR) @[ IoU=0.50:0.95 | area=medium | maxDets=100 ] = 0.571
 Average Recall     (AR) @[ IoU=0.50:0.95 | area= large | maxDets=100 ] = 0.750
Epoch: [14]  [  0/502]  eta: 0:07:08  lr: 0.000001  loss: 0.1402 (0.1402)  loss_classifier: 0.0621 (0.0621)  loss_box_reg: 0.0743 (0.0743)  loss_objectness: 0.0023 (0.0023)  loss_rpn_box_reg: 0.0016 (0.0016)  time: 0.8536  data: 0.2390  max mem: 19627
Epoch: [14]  [126/502]  eta: 0:05:18  lr: 0.000001  loss: 0.1825 (0.1999)  loss_classifier: 0.0917 (0.1030)  loss_box_reg: 0.0767 (0.0915)  loss_objectness: 0.0024 (0.0024)  loss_rpn_box_reg: 0.0020 (0.0030)  time: 0.8198  data: 0.1862  max mem: 19627
Epoch: [14]  [252/502]  eta: 0:03:30  lr: 0.000001  loss: 0.2408 (0.1989)  loss_classifier: 0.1006 (0.1021)  loss_box_reg: 0.1166 (0.0911)  loss_objectness: 0.0026 (0.0027)  loss_rpn_box_reg: 0.0027 (0.0031)  time: 0.8526  data: 0.2152  max mem: 19627
Epoch: [14]  [378/502]  eta: 0:01:44  lr: 0.000001  loss: 0.2019 (0.2031)  loss_classifier: 0.1062 (0.1050)  loss_box_reg: 0.0830 (0.0924)  loss_objectness: 0.0031 (0.0027)  loss_rpn_box_reg: 0.0021 (0.0030)  time: 0.8361  data: 0.2016  max mem: 19627
Epoch: [14]  [501/502]  eta: 0:00:00  lr: 0.000001  loss: 0.1814 (0.2037)  loss_classifier: 0.0864 (0.1055)  loss_box_reg: 0.0770 (0.0925)  loss_objectness: 0.0019 (0.0027)  loss_rpn_box_reg: 0.0020 (0.0030)  time: 0.8406  data: 0.2114  max mem: 19627
Epoch: [14] Total time: 0:07:02 (0.8420 s / it)
creating index...
index created!
Test:  [  0/642]  eta: 0:00:41  model_time: 0.0426 (0.0426)  evaluator_time: 0.0040 (0.0040)  time: 0.0654  data: 0.0148  max mem: 19627
Test:  [100/642]  eta: 0:00:33  model_time: 0.0403 (0.0406)  evaluator_time: 0.0014 (0.0017)  time: 0.0614  data: 0.0150  max mem: 19627
Test:  [200/642]  eta: 0:00:27  model_time: 0.0406 (0.0407)  evaluator_time: 0.0012 (0.0017)  time: 0.0615  data: 0.0157  max mem: 19627
Test:  [300/642]  eta: 0:00:21  model_time: 0.0409 (0.0410)  evaluator_time: 0.0012 (0.0016)  time: 0.0607  data: 0.0146  max mem: 19627
Test:  [400/642]  eta: 0:00:14  model_time: 0.0402 (0.0411)  evaluator_time: 0.0013 (0.0016)  time: 0.0614  data: 0.0145  max mem: 19627
Test:  [500/642]  eta: 0:00:08  model_time: 0.0404 (0.0411)  evaluator_time: 0.0012 (0.0018)  time: 0.0606  data: 0.0144  max mem: 19627
Test:  [600/642]  eta: 0:00:02  model_time: 0.0397 (0.0411)  evaluator_time: 0.0015 (0.0019)  time: 0.0639  data: 0.0162  max mem: 19627
Test:  [641/642]  eta: 0:00:00  model_time: 0.0413 (0.0411)  evaluator_time: 0.0016 (0.0019)  time: 0.0640  data: 0.0160  max mem: 19627
Test: Total time: 0:00:39 (0.0621 s / it)
Averaged stats: model_time: 0.0413 (0.0411)  evaluator_time: 0.0016 (0.0019)
Accumulating evaluation results...
DONE (t=0.28s).
IoU metric: bbox
 Average Precision  (AP) @[ IoU=0.50:0.95 | area=   all | maxDets=100 ] = 0.608
 Average Precision  (AP) @[ IoU=0.50      | area=   all | maxDets=100 ] = 0.825
 Average Precision  (AP) @[ IoU=0.75      | area=   all | maxDets=100 ] = 0.712
 Average Precision  (AP) @[ IoU=0.50:0.95 | area= small | maxDets=100 ] = -1.000
 Average Precision  (AP) @[ IoU=0.50:0.95 | area=medium | maxDets=100 ] = 0.443
 Average Precision  (AP) @[ IoU=0.50:0.95 | area= large | maxDets=100 ] = 0.619
 Average Recall     (AR) @[ IoU=0.50:0.95 | area=   all | maxDets=  1 ] = 0.220
 Average Recall     (AR) @[ IoU=0.50:0.95 | area=   all | maxDets= 10 ] = 0.668
 Average Recall     (AR) @[ IoU=0.50:0.95 | area=   all | maxDets=100 ] = 0.736
 Average Recall     (AR) @[ IoU=0.50:0.95 | area= small | maxDets=100 ] = -1.000
 Average Recall     (AR) @[ IoU=0.50:0.95 | area=medium | maxDets=100 ] = 0.556
 Average Recall     (AR) @[ IoU=0.50:0.95 | area= large | maxDets=100 ] = 0.748
Epoch: [15]  [  0/502]  eta: 0:06:54  lr: 0.000001  loss: 0.2627 (0.2627)  loss_classifier: 0.1601 (0.1601)  loss_box_reg: 0.0953 (0.0953)  loss_objectness: 0.0047 (0.0047)  loss_rpn_box_reg: 0.0026 (0.0026)  time: 0.8249  data: 0.2258  max mem: 19627
Epoch: [15]  [126/502]  eta: 0:05:16  lr: 0.000001  loss: 0.1410 (0.1983)  loss_classifier: 0.0795 (0.1027)  loss_box_reg: 0.0620 (0.0900)  loss_objectness: 0.0012 (0.0024)  loss_rpn_box_reg: 0.0015 (0.0032)  time: 0.8593  data: 0.2024  max mem: 19627
Epoch: [15]  [252/502]  eta: 0:03:31  lr: 0.000001  loss: 0.1602 (0.2011)  loss_classifier: 0.0672 (0.1040)  loss_box_reg: 0.0810 (0.0916)  loss_objectness: 0.0014 (0.0025)  loss_rpn_box_reg: 0.0022 (0.0031)  time: 0.8510  data: 0.2109  max mem: 19627
Epoch: [15]  [378/502]  eta: 0:01:44  lr: 0.000001  loss: 0.1768 (0.2018)  loss_classifier: 0.0926 (0.1045)  loss_box_reg: 0.0909 (0.0918)  loss_objectness: 0.0016 (0.0025)  loss_rpn_box_reg: 0.0022 (0.0030)  time: 0.8423  data: 0.2206  max mem: 19627
Epoch: [15]  [501/502]  eta: 0:00:00  lr: 0.000001  loss: 0.1981 (0.2031)  loss_classifier: 0.0979 (0.1049)  loss_box_reg: 0.0904 (0.0926)  loss_objectness: 0.0026 (0.0025)  loss_rpn_box_reg: 0.0024 (0.0030)  time: 0.8275  data: 0.2108  max mem: 19627
Epoch: [15] Total time: 0:07:02 (0.8418 s / it)
creating index...
index created!
Test:  [  0/642]  eta: 0:00:41  model_time: 0.0413 (0.0413)  evaluator_time: 0.0038 (0.0038)  time: 0.0646  data: 0.0152  max mem: 19627
Test:  [100/642]  eta: 0:00:33  model_time: 0.0407 (0.0406)  evaluator_time: 0.0013 (0.0017)  time: 0.0611  data: 0.0147  max mem: 19627
Test:  [200/642]  eta: 0:00:27  model_time: 0.0392 (0.0406)  evaluator_time: 0.0011 (0.0016)  time: 0.0603  data: 0.0154  max mem: 19627
Test:  [300/642]  eta: 0:00:21  model_time: 0.0403 (0.0408)  evaluator_time: 0.0011 (0.0016)  time: 0.0603  data: 0.0145  max mem: 19627
Test:  [400/642]  eta: 0:00:14  model_time: 0.0408 (0.0409)  evaluator_time: 0.0013 (0.0016)  time: 0.0614  data: 0.0143  max mem: 19627
Test:  [500/642]  eta: 0:00:08  model_time: 0.0402 (0.0409)  evaluator_time: 0.0013 (0.0016)  time: 0.0599  data: 0.0142  max mem: 19627
Test:  [600/642]  eta: 0:00:02  model_time: 0.0391 (0.0409)  evaluator_time: 0.0015 (0.0017)  time: 0.0633  data: 0.0160  max mem: 19627
Test:  [641/642]  eta: 0:00:00  model_time: 0.0404 (0.0409)  evaluator_time: 0.0017 (0.0017)  time: 0.0629  data: 0.0158  max mem: 19627
Test: Total time: 0:00:39 (0.0616 s / it)
Averaged stats: model_time: 0.0404 (0.0409)  evaluator_time: 0.0017 (0.0017)
Accumulating evaluation results...
DONE (t=0.28s).
IoU metric: bbox
 Average Precision  (AP) @[ IoU=0.50:0.95 | area=   all | maxDets=100 ] = 0.609
 Average Precision  (AP) @[ IoU=0.50      | area=   all | maxDets=100 ] = 0.824
 Average Precision  (AP) @[ IoU=0.75      | area=   all | maxDets=100 ] = 0.710
 Average Precision  (AP) @[ IoU=0.50:0.95 | area= small | maxDets=100 ] = -1.000
 Average Precision  (AP) @[ IoU=0.50:0.95 | area=medium | maxDets=100 ] = 0.448
 Average Precision  (AP) @[ IoU=0.50:0.95 | area= large | maxDets=100 ] = 0.620
 Average Recall     (AR) @[ IoU=0.50:0.95 | area=   all | maxDets=  1 ] = 0.221
 Average Recall     (AR) @[ IoU=0.50:0.95 | area=   all | maxDets= 10 ] = 0.668
 Average Recall     (AR) @[ IoU=0.50:0.95 | area=   all | maxDets=100 ] = 0.739
 Average Recall     (AR) @[ IoU=0.50:0.95 | area= small | maxDets=100 ] = -1.000
 Average Recall     (AR) @[ IoU=0.50:0.95 | area=medium | maxDets=100 ] = 0.580
 Average Recall     (AR) @[ IoU=0.50:0.95 | area= large | maxDets=100 ] = 0.749
Epoch: [16]  [  0/502]  eta: 0:07:23  lr: 0.000000  loss: 0.1250 (0.1250)  loss_classifier: 0.0630 (0.0630)  loss_box_reg: 0.0591 (0.0591)  loss_objectness: 0.0019 (0.0019)  loss_rpn_box_reg: 0.0010 (0.0010)  time: 0.8834  data: 0.2856  max mem: 19627
Epoch: [16]  [126/502]  eta: 0:05:09  lr: 0.000000  loss: 0.1936 (0.2073)  loss_classifier: 0.0992 (0.1057)  loss_box_reg: 0.0782 (0.0955)  loss_objectness: 0.0018 (0.0028)  loss_rpn_box_reg: 0.0022 (0.0033)  time: 0.8467  data: 0.2099  max mem: 19627
Epoch: [16]  [252/502]  eta: 0:03:27  lr: 0.000000  loss: 0.1630 (0.2018)  loss_classifier: 0.0868 (0.1028)  loss_box_reg: 0.0754 (0.0933)  loss_objectness: 0.0014 (0.0027)  loss_rpn_box_reg: 0.0015 (0.0031)  time: 0.8374  data: 0.2203  max mem: 19627
Epoch: [16]  [378/502]  eta: 0:01:43  lr: 0.000000  loss: 0.1670 (0.2014)  loss_classifier: 0.0830 (0.1034)  loss_box_reg: 0.0815 (0.0923)  loss_objectness: 0.0025 (0.0026)  loss_rpn_box_reg: 0.0020 (0.0030)  time: 0.8488  data: 0.1955  max mem: 19627
Epoch: [16]  [501/502]  eta: 0:00:00  lr: 0.000000  loss: 0.2003 (0.2014)  loss_classifier: 0.0963 (0.1035)  loss_box_reg: 0.0829 (0.0924)  loss_objectness: 0.0013 (0.0025)  loss_rpn_box_reg: 0.0023 (0.0030)  time: 0.8694  data: 0.2091  max mem: 19627
Epoch: [16] Total time: 0:06:59 (0.8365 s / it)
creating index...
index created!
Test:  [  0/642]  eta: 0:00:41  model_time: 0.0429 (0.0429)  evaluator_time: 0.0039 (0.0039)  time: 0.0652  data: 0.0144  max mem: 19627
Test:  [100/642]  eta: 0:00:33  model_time: 0.0410 (0.0408)  evaluator_time: 0.0014 (0.0017)  time: 0.0619  data: 0.0148  max mem: 19627
Test:  [200/642]  eta: 0:00:27  model_time: 0.0399 (0.0407)  evaluator_time: 0.0012 (0.0017)  time: 0.0603  data: 0.0154  max mem: 19627
Test:  [300/642]  eta: 0:00:21  model_time: 0.0401 (0.0408)  evaluator_time: 0.0011 (0.0016)  time: 0.0599  data: 0.0146  max mem: 19627
Test:  [400/642]  eta: 0:00:14  model_time: 0.0407 (0.0410)  evaluator_time: 0.0013 (0.0016)  time: 0.0617  data: 0.0144  max mem: 19627
Test:  [500/642]  eta: 0:00:08  model_time: 0.0394 (0.0409)  evaluator_time: 0.0014 (0.0018)  time: 0.0594  data: 0.0142  max mem: 19627
Test:  [600/642]  eta: 0:00:02  model_time: 0.0407 (0.0409)  evaluator_time: 0.0015 (0.0019)  time: 0.0646  data: 0.0161  max mem: 19627
Test:  [641/642]  eta: 0:00:00  model_time: 0.0406 (0.0409)  evaluator_time: 0.0017 (0.0019)  time: 0.0628  data: 0.0156  max mem: 19627
Test: Total time: 0:00:39 (0.0618 s / it)
Averaged stats: model_time: 0.0406 (0.0409)  evaluator_time: 0.0017 (0.0019)
Accumulating evaluation results...
DONE (t=0.28s).
IoU metric: bbox
 Average Precision  (AP) @[ IoU=0.50:0.95 | area=   all | maxDets=100 ] = 0.608
 Average Precision  (AP) @[ IoU=0.50      | area=   all | maxDets=100 ] = 0.823
 Average Precision  (AP) @[ IoU=0.75      | area=   all | maxDets=100 ] = 0.709
 Average Precision  (AP) @[ IoU=0.50:0.95 | area= small | maxDets=100 ] = -1.000
 Average Precision  (AP) @[ IoU=0.50:0.95 | area=medium | maxDets=100 ] = 0.449
 Average Precision  (AP) @[ IoU=0.50:0.95 | area= large | maxDets=100 ] = 0.619
 Average Recall     (AR) @[ IoU=0.50:0.95 | area=   all | maxDets=  1 ] = 0.219
 Average Recall     (AR) @[ IoU=0.50:0.95 | area=   all | maxDets= 10 ] = 0.666
 Average Recall     (AR) @[ IoU=0.50:0.95 | area=   all | maxDets=100 ] = 0.737
 Average Recall     (AR) @[ IoU=0.50:0.95 | area= small | maxDets=100 ] = -1.000
 Average Recall     (AR) @[ IoU=0.50:0.95 | area=medium | maxDets=100 ] = 0.570
 Average Recall     (AR) @[ IoU=0.50:0.95 | area= large | maxDets=100 ] = 0.749
Early stopped at epoch 16 result: 0.823 <= mean( [0.824, 0.823, 0.825, 0.824, 0.823] )
 
 
 

 

 

 
 
 
 
 
  
  
 ⚖️ Show train and validation metrics report ¶   The collected training and validation metrics report is shown below. The first four metrics about loss refer to the loss during training. The 12 metrics after that are COCO generated validation metrics over all the epochs. 

 
 
 
  
 
 
 
 
 In [ ]: 
 
      
     metrics   =   pandas  .  DataFrame  (  report  ) 
 metrics  .  plot  (  subplots  =  True  ,   layout  =  (  5  ,  4  ),   figsize  =  (  20  ,  25  ),   x  =  "epoch"  ,   sharex  =  False  ,   title  =  metrics  .  columns  .  to_list  ()[  1  :],   legend  =  False  ) 
 plt  .  subplots_adjust  (  hspace  =  0.35  ) 
  

      
 
 
 

 
 
 


 
 
    
      


 
 
 

 

 

 

 
 
 
 
 
  
  
 ✅ Run against the test data ¶   Now the model is ran against the earlier separated test dataset to get the COCO metrics per class. And again to create a confusion matrix. 

 
 
 
  
 
 
 
 
 In [ ]: 
 
      
     results  ,   metrics   =   coco_evaluator_class_metrics  (  model  ,   test_loader  ,   device  ) 
 df_testresults   =   pandas  .  DataFrame  (  results  ,   columns  =  metrics  ) 
 df_testresults  [  "Class"  ]   =   df_testresults  [  "Class"  ]  .  map  (  dict  (  enumerate  (  classes  ))) 
 df_testresults  .  drop  ([  "AP[IoU=0.50:0.95]"  ,   "AP[IoU=0.75]"  ,   "AR[IoU=0.50:0.95]"  ],   axis  =  1  ,   inplace  =  True  ) 
 df_testresults   =   df_testresults  .  sort_values  (  by  =  "AP[IoU=0.50]"  ,   ascending  =  False  ) 
 df_testresults 
  

      
 
 
 

 
 
 


 
 
    
      


 
 creating index...
index created!
Test:  [  0/630]  eta: 0:00:38  model_time: 0.0437 (0.0437)  evaluator_time: 0.0016 (0.0016)  time: 0.0617  data: 0.0130  max mem: 19627
Test:  [100/630]  eta: 0:00:32  model_time: 0.0390 (0.0401)  evaluator_time: 0.0013 (0.0016)  time: 0.0594  data: 0.0143  max mem: 19627
Test:  [200/630]  eta: 0:00:25  model_time: 0.0397 (0.0398)  evaluator_time: 0.0012 (0.0015)  time: 0.0605  data: 0.0153  max mem: 19627
Test:  [300/630]  eta: 0:00:19  model_time: 0.0401 (0.0400)  evaluator_time: 0.0012 (0.0015)  time: 0.0608  data: 0.0148  max mem: 19627
Test:  [400/630]  eta: 0:00:14  model_time: 0.0405 (0.0401)  evaluator_time: 0.0014 (0.0018)  time: 0.0624  data: 0.0154  max mem: 19627
Test:  [500/630]  eta: 0:00:07  model_time: 0.0389 (0.0402)  evaluator_time: 0.0016 (0.0018)  time: 0.0600  data: 0.0145  max mem: 19627
Test:  [600/630]  eta: 0:00:01  model_time: 0.0404 (0.0403)  evaluator_time: 0.0013 (0.0018)  time: 0.0619  data: 0.0150  max mem: 19627
Test:  [629/630]  eta: 0:00:00  model_time: 0.0403 (0.0403)  evaluator_time: 0.0025 (0.0019)  time: 0.0638  data: 0.0156  max mem: 19627
Test: Total time: 0:00:38 (0.0614 s / it)
Averaged stats: model_time: 0.0403 (0.0403)  evaluator_time: 0.0025 (0.0019)
Accumulating evaluation results...
DONE (t=0.27s).
IoU metric: bbox
 Average Precision  (AP) @[ IoU=0.50:0.95 | area=   all | maxDets=100 ] = 0.604
 Average Precision  (AP) @[ IoU=0.50      | area=   all | maxDets=100 ] = 0.821
 Average Precision  (AP) @[ IoU=0.75      | area=   all | maxDets=100 ] = 0.702
 Average Precision  (AP) @[ IoU=0.50:0.95 | area= small | maxDets=100 ] = -1.000
 Average Precision  (AP) @[ IoU=0.50:0.95 | area=medium | maxDets=100 ] = 0.479
 Average Precision  (AP) @[ IoU=0.50:0.95 | area= large | maxDets=100 ] = 0.615
 Average Recall     (AR) @[ IoU=0.50:0.95 | area=   all | maxDets=  1 ] = 0.213
 Average Recall     (AR) @[ IoU=0.50:0.95 | area=   all | maxDets= 10 ] = 0.658
 Average Recall     (AR) @[ IoU=0.50:0.95 | area=   all | maxDets=100 ] = 0.729
 Average Recall     (AR) @[ IoU=0.50:0.95 | area= small | maxDets=100 ] = -1.000
 Average Recall     (AR) @[ IoU=0.50:0.95 | area=medium | maxDets=100 ] = 0.589
 Average Recall     (AR) @[ IoU=0.50:0.95 | area= large | maxDets=100 ] = 0.744
 
 
 
 
    
     Out[ ]: 


 
 
 
 
   
     
        
       Class 
       AP[IoU=0.50] 
     
   
   
     
       33 
       Centaurea cyanus 
       0.993064 
     
     
       1 
       Ajuga reptans 
       0.981664 
     
     
       2 
       Persicaria bistorta 
       0.973919 
     
     
       18 
       Lamium album 
       0.970103 
     
     
       23 
       Lamium purpureum 
       0.969962 
     
     
       13 
       Alliaria petiolata 
       0.968566 
     
     
       8 
       Dactylorhiza praetermissa 
       0.966242 
     
     
       20 
       Cirsium arvense 
       0.961846 
     
     
       43 
       Hypericum elodes 
       0.947519 
     
     
       15 
       Anemone nemorosa 
       0.946386 
     
     
       42 
       Centaurea jacea 
       0.943478 
     
     
       48 
       Hypochaeris radicata 
       0.928032 
     
     
       21 
       Myosotis scorpioides 
       0.916769 
     
     
       28 
       Dactylorhiza maculata 
       0.905857 
     
     
       7 
       Cardamine pratensis 
       0.903207 
     
     
       11 
       Ranunculus flammula 
       0.902617 
     
     
       4 
       Silene vulgaris 
       0.899622 
     
     
       30 
       Rhinanthus angustifolius 
       0.896978 
     
     
       5 
       Silene dioica 
       0.891874 
     
     
       26 
       Caltha palustris 
       0.890985 
     
     
       19 
       Erodium cicutarium 
       0.888660 
     
     
       9 
       Papaver rhoeas 
       0.884813 
     
     
       47 
       Dianthus deltoides 
       0.874834 
     
     
       35 
       Berteroa incana 
       0.874488 
     
     
       40 
       Crepis capillaris 
       0.869465 
     
     
       38 
       Bellis perennis 
       0.869007 
     
     
       14 
       Trifolium pratense 
       0.858624 
     
     
       24 
       Dianthus carthusianorum 
       0.855494 
     
     
       34 
       Ficaria verna 
       0.851149 
     
     
       41 
       Cerastium arvense 
       0.848121 
     
     
       27 
       Eupatorium cannabinum 
       0.821500 
     
     
       31 
       Aegopodium podagraria 
       0.814003 
     
     
       36 
       Hieracium umbellatum 
       0.813991 
     
     
       17 
       Anthyllis vulneraria 
       0.796470 
     
     
       16 
       Achillea millefolium 
       0.781735 
     
     
       46 
       Pedicularis palustris 
       0.768939 
     
     
       37 
       Daucus carota 
       0.739878 
     
     
       12 
       Tanacetum vulgare 
       0.737947 
     
     
       10 
       Plantago lanceolata 
       0.733133 
     
     
       39 
       Chamomile * (aggregate) 
       0.726392 
     
     
       29 
       Leucanthemum vulgare 
       0.713738 
     
     
       3 
       Lotus corniculatus 
       0.692151 
     
     
       25 
       Vicia cracca 
       0.651708 
     
     
       0 
       Trifolium repens 
       0.642630 
     
     
       45 
       Buttercup * (aggregate) 
       0.589454 
     
     
       44 
       Glechoma hederacea 
       0.584965 
     
     
       32 
       Anchusa officinalis 
       0.523763 
     
     
       22 
       Ranunculus aquatilis 
       0.447706 
     
     
       6 
       Anthriscus sylvestris 
       0.191667 
     
   
 
 
 

 

 

 

  
 
 
 
 
 In [ ]: 
 
      
     plt  .  figure  (  figsize  =  (  15  ,   4  ))  
 plot   =   seaborn  .  barplot  (  x  =  df_testresults  [  "Class"  ],   y  =  df_testresults  [  "AP[IoU=0.50]"  ]) 
 plot  .  bar_label  (  plot  .  containers  [  0  ],   rotation  =  "vertical"  ,   fmt  =  "  %.3f  "  ,   padding  =-  29  ) 
 plot  .  tick_params  (  axis  =  "x"  ,   labelrotation  =  90  ) 
 plot 
  

      
 
 
 

 
 
 


 
 
    
     Out[ ]: 


 
 <AxesSubplot: xlabel='Class', ylabel='AP[IoU=0.50]'> 
 

 
 
    
      


 
 
 

 

 

 

  
 
 
 
 
 In [ ]: 
 
      
     def   generate_confusion_matrix  (  model  ,   device  ,   dataset  ,   classes  ,   score_threshold  =  .7  ,   iou_threshold  =  .5  ): 
     import   torchvision.ops.boxes   as   bops 
     num_threads   =   torch  .  get_num_threads  () 
     torch  .  set_num_threads  (  1  ) 
     cpu_device   =   torch  .  device  (  "cpu"  ) 
     model   =   model  .  to  (  device  ) 
     model  .  eval  () 
     matrix_size   =   len  (  classes  ) 
     matrix   =   [[  0   for   x   in   range  (  matrix_size  )]   for   y   in   range  (  matrix_size  )] 
    
     for   i   in   range  (  len  (  dataset  )): 
         img  ,   targets   =   dataset  [  i  ] 
         imgs   =   list  ([  img  .  to  (  device  )]) 
         if   torch  .  cuda  .  is_available  (): 
             torch  .  cuda  .  synchronize  () 
         output   =   model  (  imgs  ) 
         output  [  0  ][  "boxes"  ]   =   output  [  0  ][  "boxes"  ][  output  [  0  ][  "scores"  ]   >=   score_threshold  ] 
         output  [  0  ][  "labels"  ]   =   output  [  0  ][  "labels"  ][  output  [  0  ][  "scores"  ]   >=   score_threshold  ] 
         output  [  0  ][  "scores"  ]   =   output  [  0  ][  "scores"  ][  output  [  0  ][  "scores"  ]   >=   score_threshold  ] 
         output   =   [{  k  :   v  .  to  (  cpu_device  )   for   k  ,   v   in   t  .  items  ()}   for   t   in   output  ] 

         output   =   output  [  0  ] 
         pred_boxes   =   output  [  "boxes"  ]  .  detach  () 
         pred_labels   =   output  [  "labels"  ]  .  detach  ()  .  tolist  () 
         truth_boxes   =   targets  [  "boxes"  ]  .  detach  () 
         truth_labels   =   targets  [  "labels"  ]  .  detach  ()  .  tolist  () 

         ious   =   bops  .  box_iou  (  truth_boxes  ,   pred_boxes  )  .  detach  () 
         for   tid  ,   v   in   enumerate  (  ious  ): 
             if   len  (  v  )   ==   0   or   torch  .  max  (  v  )   <   iou_threshold  :   # missed truth box 
                 q   =   truth_labels  [  tid  ] 
                 matrix  [  truth_labels  [  tid  ]][  0  ]   +=   1 
             else  :   # overlaps 
                 pid   =   torch  .  topk  (  v  ,   1  )[  1  ]  .  item  () 
                 tindex   =   truth_labels  [  tid  ] 
                 pindex   =   pred_labels  [  pid  ] 
                 matrix  [  tindex  ][  pindex  ]   +=   1 
         ious   =   bops  .  box_iou  (  pred_boxes  ,   truth_boxes  )  .  detach  () 
         for   pid  ,   v   in   enumerate  (  ious  ): 
             pindex   =   pred_labels  [  pid  ] 
             if   torch  .  sum  (  v  )  .  item  ()   ==   0  :   # prediction with no matching truth box. 
                 matrix  [  0  ][  pindex  ]   +=   1 

     torch  .  set_num_threads  (  num_threads  ) 
     return   matrix 

 matrix   =   generate_confusion_matrix  (  model  ,   device  ,   test_dataset  ,   classes  ) 
 plt  .  figure  (  figsize  =  (  15  ,  15  )) 
 seaborn  .  heatmap  (  matrix  ,   annot  =  True  ,   xticklabels  =  classes  ,   yticklabels  =  classes  ,   cmap  =  "Blues"  ) 
  

      
 
 
 

 
 
 


 
 
    
     Out[ ]: 


 
 <AxesSubplot: > 
 

 
 
    
      


 
 
 

 

 

 

  
 
 
 
 
 In [ ]: 
 
      
     matrix   =   generate_confusion_matrix  (  model  ,   device  ,   test_dataset  ,   classes  ,  score_threshold  =  .5  ) 
 plt  .  figure  (  figsize  =  (  15  ,  15  )) 
 seaborn  .  heatmap  (  matrix  ,   annot  =  True  ,   xticklabels  =  classes  ,   yticklabels  =  classes  ,   cmap  =  "Blues"  ) 
  

      
 
 
 

 
 
 


 
 
    
     Out[ ]: 


 
 <AxesSubplot: > 
 

 
 
    
      


 
 
 

 

 

 

 
 
 
 
 
  
  
 💾 Save the model ¶   The model is saved to disk using the pytorch default  model.state_dict()  that produces a file can be loaded again later. 

 
 
 
  
 
 
 
 
 In [ ]: 
 
      
     torch  .  save  (  model  .  state_dict  (),   os  .  path  .  join  (  output_path  ,   "model"  )) 
 f   =   open  (  os  .  path  .  join  (  output_path  ,   "classes.json"  ),   'w'  ) 
 json  .  dump  (  classes  ,   f  ) 
 f  .  close  () 
  

      
 
 
 

 
 


 
