# Supplementary material for: Data-centric AI approach for automated wildflower monitoring
Source: PLoS One. 2024 Sep 9;19(9):e0302958. doi: 10.1371/journal.pone.0302958 (PMC11383241; doi:10.1371/journal.pone.0302958)
Supplement: S2 File — (HTML) [file pone.0302958.s003.html]

inference


In [1]:

```
import json, os

import matplotlib.pyplot as plt
import torchvision.models.detection as models
import torchvision.transforms.functional as transforms

import torch
print("Torch version:", torch.__version__) # 0.13.1+cu116

import objectdetectiontools
print("Object Detection Tools version:", objectdetectiontools.__version__) # 1.2.6
```

```
Torch version: 1.13.0
Object Detection Tools version: 1.2.6
```

# Input parameters¶

In order to run this notebook the following parameters need to be provided:

- `model_path` the path to the saved model to use. The notebook `flowerpower.ipynb` can be used to create models.
- `image_path` the path to the image to run object detection on.
- `output_dir` the destination directory for the output images. Will be created if it does not exist yet.
- `threshold` the minimum accuracy for a detection to be drawn as a bounding box in the output file.
- `font_file` the filename of the font to use for the labels in the output file. Will look in the default OS font directory.
- `font-size` the size of the font to use for the labels in the output file.
- `line-width` the width of the line used to draw bounding boxes in the output file.
- `color` the color of the line used to draw bounding boxes, as well as for the font used for the labels in the output file.

In [2]:

```
model_path = "../models/frcnn_20240416"
image_path = "../images/_B4A6486.JPG"
output_dir = "../inferences"

threshold = .6

font_file = "Arial.ttf"
font_size = 18
line_width = 3
color = "#FF00DC"
```

In [3]:

```
if model_path == "" or image_path == "" or output_dir == "":
    raise Exception("None of the path parameters can be empty, please provide a value for all of them")
if threshold > 1 or threshold < .01:
    raise Exception("Parameter 'threshold' cannot be larger than 1 or smaller than .01")
if font_file == "":
    raise Exception("Parameter 'font_file' cannot be empty")
if font_size < 1:
    raise Exception("Parameter 'font_size' cannot be smaller than 1")
if line_width < 1:
    raise Exception("Parameter 'line_width' cannot be smaller than 1")
if color == "":
    raise Exception("Parameter 'color' cannot be empty")
```

# 📥 Loading the model¶

Loading the model from a previously saved state.

In [4]:

```
f = open(os.path.join(model_path, "classes.json"))
classes = json.load(f)
f.close()
model = models.fasterrcnn_resnet50_fpn_v2(weights="DEFAULT")
model.roi_heads.box_predictor = models.faster_rcnn.FastRCNNPredictor(model.roi_heads.box_predictor.cls_score.in_features, len(classes))
device = torch.device("cuda" if torch.cuda.is_available() else "cpu")
state = torch.load(f=os.path.join(model_path, "model"), map_location=device)
model.load_state_dict(state_dict=state)
model.eval()
print("Loaded a model of type", str(type(model).__name__), "for", len(classes), "classes on", device)
print("The following classes are known:")
print(classes)
```

```
Loaded a model of type FasterRCNN for 50 classes on cpu
The following classes are known:
['__background__', 'Trifolium repens', 'Ajuga reptans', 'Persicaria bistorta', 'Lotus corniculatus ', 'Silene vulgaris', 'Silene dioica', 'Anthriscus sylvestris', 'Cardamine pratensis', 'Dactylorhiza praetermissa', 'Papaver rhoeas', 'Plantago lanceolata', 'Ranunculus flammula', 'Tanacetum vulgare', 'Alliaria petiolata', 'Trifolium pratense', 'Anemone nemorosa', 'Achillea millefolium', 'Anthyllis vulneraria', 'Lamium album', 'Erodium cicutarium', 'Cirsium arvense', 'Myosotis scorpioides', 'Ranunculus aquatilis', 'Lamium purpureum', 'Dianthus carthusianorum', 'Vicia cracca', 'Caltha palustris', 'Eupatorium cannabinum', 'Dactylorhiza maculata', 'Leucanthemum vulgare', 'Rhinanthus angustifolius', 'Aegopodium podagraria', 'Anchusa officinalis', 'Centaurea cyanus', 'Ficaria verna', 'Berteroa incana', 'Hieracium umbellatum', 'Daucus carota', 'Bellis perennis', 'Chamomile * (aggregate)', 'Crepis capillaris', 'Cerastium arvense', 'Centaurea jacea', 'Hypericum elodes', 'Glechoma hederacea', 'Buttercup * (aggregate)', 'Pedicularis palustris', 'Dianthus deltoides', 'Hypochaeris radicata']
```

# 🎴 Preprocessing¶

Cut the input image into tiles.

In [5]:

```
tiles, rows, columns = objectdetectiontools.images.chop(image_path, model.transform.max_size)
fig, subplots = plt.subplots(nrows=rows, ncols=columns, figsize=(5*columns, 5*rows))
subplots = subplots.flatten()
for i, t in enumerate(tiles):
    subplots[i].set_xticks([])
    subplots[i].set_yticks([])
    subplots[i].imshow(t)
```

# 🔎 Inference¶

Run a prediction on every tile from the previous step.

In [6]:

```
def inference(model, image, threshold):
    tensor = transforms.pil_to_tensor(image).unsqueeze(dim=0) / 255
    predictions = model(tensor)
    predictions[0]["boxes"] = predictions[0]["boxes"][predictions[0]["scores"] > threshold]
    predictions[0]["labels"] = predictions[0]["labels"][predictions[0]["scores"] > threshold]
    predictions[0]["scores"] = predictions[0]["scores"][predictions[0]["scores"] > threshold]
    return predictions

def add_bounding_boxes(image, classes, predictions, font_size, line_width, color):
    from torchvision.utils import draw_bounding_boxes
    annotations = []
    for p in predictions[0]["labels"].detach().numpy():
        annotations.append({"name": classes[p]})
    labels = ["{} {}%".format(label["name"], int(prob *100)) for label, prob in zip(annotations, predictions[0]["scores"].detach().numpy())]
    output = draw_bounding_boxes(image=transforms.pil_to_tensor(image).unsqueeze(dim=0)[0], boxes=predictions[0]["boxes"], labels=labels, colors=color, width=line_width, font=font_file, font_size=font_size)
    return transforms.to_pil_image(output) 

print("Running inference for", len(tiles), "tiles:")
fig, subplots = plt.subplots(nrows=rows, ncols=columns, figsize=(5*columns, 5*rows))
subplots = subplots.flatten()
result_images = []
result_predictions = []
for index, tile in enumerate(tiles):
    print("Tile", index+1)
    predictions = inference(model, tile, threshold)
    result_predictions.append(predictions)
    result_image = add_bounding_boxes(tile, classes, predictions, font_size, line_width, color) if len(predictions[0]["boxes"]) > 0 else tile
    result_images.append(result_image)
    subplots[index].set_xticks([])
    subplots[index].set_yticks([])
    subplots[index].imshow(result_image)
```

```
Running inference for 15 tiles:
Tile 1
Tile 2
Tile 3
Tile 4
Tile 5
Tile 6
Tile 7
Tile 8
Tile 9
Tile 10
Tile 11
Tile 12
Tile 13
Tile 14
Tile 15
```

# 🪡 Stitch¶

Stitch the tiles back together again and save the result.

In [7]:

```
if not os.path.exists(output_dir):
    os.mkdir(output_dir)
output_file = os.path.join(output_dir, os.path.basename(image_path))
result = objectdetectiontools.images.stitch(result_images, columns)
result.save(output_file)
print("Inference image saved at", output_file)
```

```
Inference image saved at ../inferences/_B4A6486.JPG
```

# 🧮 Counting¶

The code below generates an overview of the found labels and the number of times they appear on the image.

In [8]:

```
countings = dict()
for prediction in result_predictions:
    for label in prediction[0]["labels"].detach().numpy():
        label = classes[label]
        countings[label] = countings[label] +1 if label in countings else 1

for key, value in countings.items():
    print(key, ":", value)
```

```
Dactylorhiza praetermissa : 19
Buttercup * (aggregate) : 16
```
